# Globally doubled methane emissions from nutrient-enriched rivers

**DOI:** 10.1093/nsr/nwag192

**Published:** 2026-03-26

**Authors:** Junfeng Wang, Xinghui Xia, Shaoda Liu, Sibo Zhang, Junyu Dong, Gongqin Wang, Wenhao Xu, Ling Zhang, Wenxiu Zheng, Zhuangzhuang Zhang, Xin Chen, Linfeng Yuan, Jiao Liu, Jiajia Zhang, Yuan Xin, William H McDowell, Hanqin Tian, David Bastviken, Zhifeng Yang

**Affiliations:** Key Laboratory of Water and Sediment Sciences of Ministry of Education, State Key Laboratory of Wetland Conservation and Restoration, School of Environment, Beijing Normal University, Beijing 100875, China; Key Laboratory of Water and Sediment Sciences of Ministry of Education, State Key Laboratory of Wetland Conservation and Restoration, School of Environment, Beijing Normal University, Beijing 100875, China; Key Laboratory of Water and Sediment Sciences of Ministry of Education, State Key Laboratory of Wetland Conservation and Restoration, School of Environment, Beijing Normal University, Beijing 100875, China; Guangdong Provincial Key Laboratory of Water Quality Improvement and Ecological Restoration for Watersheds, Institute of Environmental and Ecological Engineering, Guangdong University of Technology, Guangzhou 510006, China; Key Laboratory of Water and Sediment Sciences of Ministry of Education, State Key Laboratory of Wetland Conservation and Restoration, School of Environment, Beijing Normal University, Beijing 100875, China; Hebei Key Laboratory of Close-to-Nature Restoration Technology of Wetlands, School of Eco-Environment, Hebei University, Baoding 071002, China; Key Laboratory of Water and Sediment Sciences of Ministry of Education, State Key Laboratory of Wetland Conservation and Restoration, School of Environment, Beijing Normal University, Beijing 100875, China; Key Laboratory of Lower Yellow River Channel and Estuary Regulation, Yellow River Institute of Hydraulic Research, Zhengzhou 450003, China; Key Laboratory of Water and Sediment Sciences of Ministry of Education, State Key Laboratory of Wetland Conservation and Restoration, School of Environment, Beijing Normal University, Beijing 100875, China; Key Laboratory of Water and Sediment Sciences of Ministry of Education, State Key Laboratory of Wetland Conservation and Restoration, School of Environment, Beijing Normal University, Beijing 100875, China; Key Laboratory of Water and Sediment Sciences of Ministry of Education, State Key Laboratory of Wetland Conservation and Restoration, School of Environment, Beijing Normal University, Beijing 100875, China; Key Laboratory of Water and Sediment Sciences of Ministry of Education, State Key Laboratory of Wetland Conservation and Restoration, School of Environment, Beijing Normal University, Beijing 100875, China; Key Laboratory of Water and Sediment Sciences of Ministry of Education, State Key Laboratory of Wetland Conservation and Restoration, School of Environment, Beijing Normal University, Beijing 100875, China; Key Laboratory of Water and Sediment Sciences of Ministry of Education, State Key Laboratory of Wetland Conservation and Restoration, School of Environment, Beijing Normal University, Beijing 100875, China; Key Laboratory of Water and Sediment Sciences of Ministry of Education, State Key Laboratory of Wetland Conservation and Restoration, School of Environment, Beijing Normal University, Beijing 100875, China; Department of Natural Resources and the Environment, University of New Hampshire, Durham 03824, USA; Department of Earth and Environmental Sciences, Schiller Institute for Integrated Science and Society, Boston College, Chestnut Hill 02467, USA; Department of Thematic Studies–Environmental Change, Linköping University, Linköping 581 83, Sweden; Guangdong Provincial Key Laboratory of Water Quality Improvement and Ecological Restoration for Watersheds, Institute of Environmental and Ecological Engineering, Guangdong University of Technology, Guangzhou 510006, China

**Keywords:** methane emission and mitigation, nutrient enrichment, river, greenhouse gas, anthropogenic impact

## Abstract

Methane (CH_4_) accounts for about one-third of the current anthropogenic greenhouse gas-driven warming. Rivers, particularly those draining human-impacted landscapes, are important sources of CH_4_ to the atmosphere. Yet, effective mitigation of this important flux remains elusive due to the lack of accurate quantification of human-induced emissions and a poor understanding of its key aquatic drivers. Here, we demonstrate that CH_4_ emission rates from global nutrient-enriched rivers in human-impacted regions more than double (2.5-fold) those from pristine rivers. Importantly, a strong association is identified between CH_4_ flux and concentrations of total phosphorus and ammonium nitrogen in these systems. This relationship is mediated by increased autochthonous and exogenous supplies of labile organic substrates, enhanced methanogen abundance and diversity, and extended anoxia in nutrient-enriched rivers. Quantitative modeling incorporating nutrient effects estimates global CH_4_ emissions from nutrient-enriched rivers at 9.7 ± 1.1 Tg CH_4_ yr^−1^, of which 22%–49% could be mitigated by reducing current nutrient concentrations to levels constrained by sustainable development goals, half of their current levels, or pristine conditions. These findings highlight the substantial potential for effective mitigation of riverine CH_4_ emissions via coordinated riverine nutrient management at the global scale.

## Teaser

This study demonstrates that anthropogenic nutrient enrichment has amplified global riverine methane emissions by fueling microbial methane production. Reducing nutrient inputs could mitigate these emissions by up to ∼50%, emphasizing the role of river nutrient management as a critical climate mitigation strategy.

## INTRODUCTION

Methane (CH_4_) is a potent greenhouse gas (GHG), accounting for about one-third of the current anthropogenic GHG-driven warming [1]. Though rivers and streams occupy a small fraction of the Earth’s land surface area, they play an essential role in the global CH_4_ budget by receiving and converting large amounts of terrestrial carbon (C) to CH_4_ [2–4]. Recent research has advanced our understanding of the importance of watershed-scale land-river connectivity in regulating the supply of CH_4_ and therefore evasion of the gas to the atmosphere from river surfaces [5–7]. Building on these insights, efforts have leveraged watershed properties—including biological, climatic, edaphic, geomorphological factors—to improve the global estimate of river CH_4_ emissions [5]. However, these watershed-scale characteristics exert only indirect, distal controls on fluvial CH_4_ emissions, making earlier estimates lacking a strong mechanistic basis and therefore highly uncertain. Crucially, *in situ* aquatic CH_4_ production has been demonstrated to contribute substantially to river CH_4_ emissions [8–10]. Aquatic conditions exert more direct controls on these emissions, particularly where

limited hydrologic connectivity restricts lateral inputs of CH_4_ and organic substrates [11]. Nevertheless, the global aquatic controls on river CH_4_ emissions remain unresolved and large-scale efforts linking aquatic factors to emission rates have been scarce. This knowledge gap not only introduces substantial uncertainty in global fluvial CH_4_ emission estimates but also significantly impedes our understanding of the controls and mitigation of the emission.

Earlier local and regional studies have highlighted the close associations between river CH_4_ fluxes and multiple aquatic factors [12–15], such as dissolved oxygen (DO), water temperature, and dissolved organic carbon (DOC), suggesting the critical controls exerted by aquatic conditions on both the magnitude and spatial patterns of CH_4_ emissions from rivers [16]. Importantly, macronutrients—primarily nitrogen (N) and phosphorus (P)—are fundamental to aquatic life and serve as sensitive indicators of anthropogenic pressures on watersheds, profoundly shaping the biogeochemical functioning of global freshwater ecosystems [17,18]. Their widespread enrichment since the onset of the Anthropocene has triggered profound ecological disruptions in freshwater systems globally [19–21]. Although macronutrient concentrations have been suggested to affect river CH_4_ fluxes [22–25], current insights remain largely tethered to localized studies with limited variability in aquatic conditions. The extent and underlying mechanism of their impacts across diverse environments and varying spatial scales have yet to be revealed [26]. Consequently, the absence of a robust, globally generalized linkage significantly hampers the accurate quantification of CH_4_ emissions driven by anthropogenic nutrient enrichment. Given that accelerating urbanization and agricultural intensification are projected to further exacerbate water deterioration [19], resolving these knowledge gaps is paramount for developing a unified framework to understand both global patterns of fluvial CH_4_ emissions under anthropogenic forcing and fully constrain humanity’s perturbation of this critical climate process.

Here, to explore key aquatic controls, in particular the impacts of aquatic anthropogenic nutrient enrichment on river CH_4_ emissions at the global scale, two complementary datasets were used: (1) a systematic 5-year field survey of China’s human-impacted rivers, and (2) the updated global River Methane Database (GRiMeDB), encompassing diverse river systems worldwide [27]. Our analysis demonstrates that CH_4_ emission rates from nutrient-enriched rivers draining human-impacted landscapes (i.e. urban settlements, croplands, or population density >20 people km^−2^) are more than 2-fold higher than those from pristine rivers. Significantly strengthened coupling is observed between nutrient concentrations and CH_4_ emissions in nutrient-enriched rivers, with total phosphorus (TP) and ammonium nitrogen (NH_4_^+^-N) emerging as the strongest predictors, suggesting that nutrient enrichment serves as a crucial control driving elevated CH_4_ emissions in these systems. By leveraging the strong predictive power of aquatic nutrients and other environmental variables, our upscaling result demonstrates that anthropogenic nutrient enrichment drives half of CH_4_ emissions from nutrient-enriched rivers globally, which is theoretically mitigable through nutrient control. These findings underscore targeted efforts to mitigate anthropogenic nutrient enrichment have co-benefits for water quality improvement and GHG mitigation.

## RESULTS AND DISCUSSION

### High CH_4_ emissions from Chinese nutrient-enriched rivers

To explore the impacts of aquatic variables on riverine CH_4_ emissions, correlations between eight aquatic variables and CH_4_ concentration and flux were firstly tested using our Chinese field data (Fig. S1). A primary comparative analysis with the GRiMeDB [27] suggested substantially elevated nutrient concentrations in these Chinese rivers, with median TP, NH_4_^+^-N, total nitrogen (TN) and nitrate nitrogen (NO_3_^−^-N) concentrations 5–36 times higher than those of the GRiMeDB (*P* < 0.001, Wilcoxon rank-sum test, Fig. S2). These Chinese rivers also exhibited 10-fold higher median chlorophyll *a* concentration (*P* < 0.001, Fig. S2), indicating significantly stronger eutrophication than global counterparts included in the GRiMeDB. Median CH_4_ emission rates were more than twice those from northern temperate rivers draining relatively pristine landscapes (*P* < 0.05, Fig. S3), suggesting elevated CH_4_ emissions from Chinese nutrient-enriched rivers.

Among the aquatic variables, five (TP, NH_4_^+^-N, DOC, DO, and water temperature) exhibited significant correlations with CH_4_ concentration and diffusive flux, with standardized regression coefficients (in absolute values, same below; see Materials and Methods) ranging from 0.15 to 0.54 (*P* < 0.001–0.01, Fig. S1b). These correlations were comparable to and/or stronger than those with terrestrial upland predictors previously used for scaling CH_4_ emissions from global rivers (0–0.28, Fig. S4) [5], indicating the importance of *in situ* aquatic conditions in dictating CH_4_ emissions from rivers. Disregarding these conditions would likely introduce substantial bias to emission estimates. Notably, TP and NH_4_^+^-N demonstrated the strongest positive correlations with both CH_4_ concentration (0.41–0.54, *P* < 0.001) and diffusive flux (0.43–0.54, *P* < 0.001, Fig. S1b) among the variables, identifying them as the primary predictors of CH_4_ emissions in Chinese nutrient-enriched rivers. Their correlations were much stronger than those of DOC (the conventional proxy for methanogenic substrate availability in rivers) which is typically regarded as a main regulator of CH_4_ emissions in prior studies [13,28]. In contrast, neither TN nor NO_3_^−^-N concentrations exhibited significant relationships with CH_4_ concentration and flux (*P* > 0.05, Fig. S1b), suggesting TN and NO_3_^−^-N likely exerted a disparate effect on aquatic CH_4_ production and emission compared to TP and NH_4_^+^-N.

### Nutrient enrichment enhances CH_4_ emissions from global human-impacted rivers

**Figure 1.**
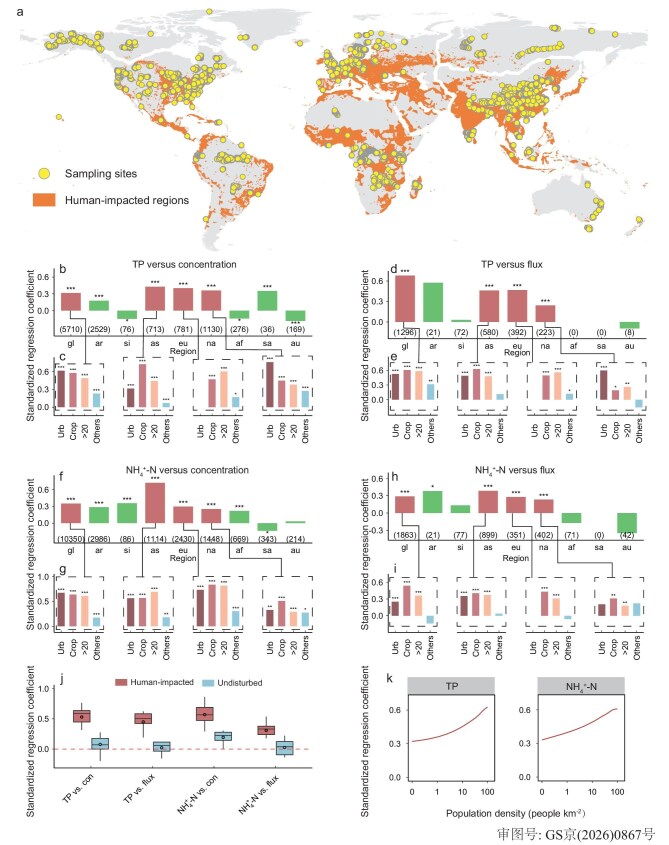
Relationships between TP and NH_4_^+^-N and CH_4_ concentrations and fluxes across global datasets. (a) Geographical distributions of sampling sites of global river networks and the extent of human-impacted area. (b–i) Standardized regression coefficients between TP and NH_4_^+^-N and CH_4_ concentration and fluxes in rivers across continents (b, d, f, h) and disturbance types (c, e, g, i). (j) Standardized regression coefficients between TP and NH_4_^+^-N and CH_4_ concentration and fluxes across human-impacted and undisturbed subdatasets. (k) Standardized regression coefficients between TP and NH_4_^+^-N and CH_4_ concentration and fluxes at different population densities. Human-impacted rivers refer to rivers situated in areas impacted by human activities, including urban settlements, croplands, and densely populated (i.e. >20 people km^−2^) regions. All variables were standardized using the Z-score normalization method before standardized linear regressions. (b, d, f, h) Subdatasets across different continents: gl, global; ar, Alaska; si, Siberia; as, Asia; eu, Europe; na, North America; af, Africa; sa, South America; au, Australia. (c, e, g, i) Sub-datasets across different disturbance types: Urb, urban; Crop, croplands; >20, population density >20 people km^−2^; Others, subdatasets excluding foregoing three disturbance types (i.e. undisturbed landscapes). Figures below the bars in panels (b, d, f, h) represent the sample size used in the regression analysis, and the asterisks above the bars indicate the statistical significance of the regression results. Data in each box of (j) include standardized regression coefficients from human-impacted and undisturbed subdatasets. Box spans the 25th and 75th percentiles. Solid line denotes the median and the whiskers represent 1.5× the interquartile range. Statistical significance between groups was tested with the two-sided Wilcoxon rank-sum test. Significance levels are represented as follows: ***P < 0.001; **P < 0.01; *P < 0.05. The curves of the standardized regression coefficients in panel (k) have been smoothed using loess method. The base map in (a) is applied from the Database of Global Administrative Areas (https://gadm.org/).

The updated GRiMeDB, with measurements covering all major continents (see Materials and Methods; Fig. 1), was used to test whether similar impacts of aquatic nutrient enrichment on CH_4_ concentration or flux can be observed in rivers from other global regions. We also observed significantly higher CH_4_ emissions from rivers in human-impacted regions (i.e. urban settlements, croplands, or landscapes with population density >20 people km^−2^, see Supplementary Materials), with mean emission rates ∼2.5-fold of those in pristine rivers (Fig. S5). These human-impacted river are also nutrient-enriched, with median nutrient concentrations (TP, NH_4_^+^-N, TN and NO_3_^−^-N) 5–42 times higher than those in pristine rivers (Fig. S6). While TP and NH_4_^+^-N, the two nutrient species most strongly correlated with CH_4_ concentration and flux in Chinese rivers (Fig. S1b), did not exhibit the same strong and positive correlations with CH_4_ concentration or flux across all continents (Fig. 1b, d, f and h). The divergence was however most pronounced in rivers draining large portions (>70%–90%) of pristine landscapes in high-latitude or tropical regions (e.g. Siberia, Alaska, or Amazonia; Fig. 1b, d, f and h), though earlier research also indicates elevated CH_4_ emissions from rivers in these regions [7,29,30]. Corresponding measurements revealed low nutrient concentrations in these rivers (e.g. median values correspond to only 0.8%–4.6% of those observed in the Chinese nutrient-enriched rivers), suggesting potential alternative drivers of high CH_4_ emissions from rivers draining these landscapes [5,31].

In contrast, TP and NH_4_^+^-N were positively correlated with CH_4_ concentration and flux in the three developed, northern continents—Asia, Europe, and North America (0.23–0.72, *P* < 0.001)—and in the global dataset (0.29–0.68, *P* < 0.001; Fig. 1 b, d, f and h), indicating a clearer signal of the nutrient effects on CH_4_ emissions from rivers in these regions. Varying fractions of the measurements (7%–53%) were taken from rivers draining relatively pristine landscapes in the three developed continents (Fig. 1a), with their median nutrient concentrations only 2.4%–27% of those found in rivers draining corresponding human-impacted landscapes in these regions (Fig. S6). When constraining the analysis to rivers draining urban settlements, croplands, or landscapes with population density >20 people km^−2^ (Fig. 1a), correlations with TP and NH_4_^+^-N became significantly stronger than those observed in the continental sub-datasets (median correlation coefficient: 0.47 versus 0.37, *P* < 0.01, Wilcoxon rank-sum test; Fig. 1c, e, g and i), suggesting a strengthening of nutrient effects in rivers draining human-impacted landscapes. In addition, these nutrient effects remained persistently strong across all temperature groups (Fig. S7), signifying that their influence on CH_4_ emissions is decoupled from temperature variation. In contrast, corresponding correlations in rivers draining the rest of pristine landscapes became significantly weaker or even negative (−0.16 to 0.30), pointing to divergent nutrient impacts similar to those observed in high-latitude or tropical rivers (Fig. 1c, e, g and i).

The strengthening in correlations with TP and NH_4_^+^-N in nutrient-enriched rivers became even more apparent when all the above identified human-impacted rivers were pooled together (Fig. 1j), with rivers draining human-impacted landscapes showing 5–23 times higher median nutrient concentrations (Fig. S6), and significantly stronger correlations with both TP (median correlation coefficient: 0.52–0.60 versus 0.06–0.12; *P* < 0.001; Wilcoxon rank-sum test) and NH_4_^+^-N concentrations (median correlation coefficient: 0.35–0.59 versus 0.04–0.18; *P* < 0.001; Wilcoxon rank-sum test) than those draining corresponding pristine landscapes (Fig. 1j). This effect was further substantiated by the strengthening correlations with concentrations of the two nutrient species in rivers draining landscapes along an increasing population density gradient (Fig. 1k), as these landscapes were associated with progressively higher TP and NH_4_^+^-N concentrations in rivers at the same time (Fig. S8).

### Underlying mechanisms linking nutrients to riverine CH_4_ emissions

The strong positive correlations with aquatic TP and NH_4_^+^-N concentrations point to a linkage between elevated CH_4_ emissions and anthropogenically driven nutrient and carbon enrichment in human-impacted rivers. We propose that nutrient enrichment amplifies CH_4_ emissions primarily by enhancing aquatic autotrophy and increasing the supply of labile autochthonous organic matter. Existing research demonstrates that labile autochthonous carbon derived from primary production is critical for CH_4_ emissions in aquatic systems [32,33], as it is more readily metabolized by methanogens than recalcitrant terrestrial carbon [34]. This connection is corroborated by the positive correlations between CH_4_ concentrations/fluxes and chlorophyll *a* concentration (a proxy for primary productivity in aquatic systems) in Chinese and the global datasets (Fig. S9), aligning with findings from other studies [35,36].

In addition, elevated nutrient concentrations highly co-occur with multiple processes in human-impacted rivers—including exogenous organic matter inputs from wastewater effluents and agricultural runoff [12,37,38], enhanced methanogen abundance and diversity [24], and the extended anoxia [39]—that collectively create favorable conditions for CH_4_ production. Domestic wastewater constitutes a major source of organic matter to rivers, especially pronounced in densely populated regions [40]. Nutrient-rich discharges introduce concentrated loads of low-molecular-weight labile organic compounds that are highly amenable to microbial decomposition [41], rapidly depleting oxygen and promoting methanogenesis [42]. Therefore, we suggest that nutrient concentrations act as a composite driver that integrates these co-occurring processes, creating a synergistic set of conditions highly favorable for methanogenesis (Fig. S10). Our field observations provided direct evidence that elevated nutrient levels promoted both abundance and diversity of methanogens in sediments (Fig. S11), leading to elevated porewater CH_4_ concentration (Fig. S11). This suggests a direct microbial linkage between nutrient enrichment and sediment methanogenesis.

In contrast, the much weaker or divergent correlations with TN (0.03–0.48, median correlation coefficient: 0.24) and NO_3_^−^-N (0–0.35, median correlation coefficient: 0.16, Fig. S12) in human-impacted rivers (despite overall increases in their correlation coefficients compared to those in pristine rivers, Fig. S13a, b) or along the population density gradient (Fig. S13c, d) were however suggested to result from the dual effects of NO_3_^−^-N on aquatic CH_4_ production [43]. Besides stimulating CH_4_ emissions as a macronutrient, NO_3_^−^-N also functions as an effective oxidizer that consumes CH_4_ in rivers with high NO_3_^−^-N concentrations [14,44,45]. Nonetheless, significantly higher CH_4_ emissions from the identified human-impacted rivers compared to those from pristine rivers (Fig. S5) suggested that the inhibitory effects of NO_3_^−^-N are likely limited compared to the stimulating effects of anthropogenically derived nutrient inputs on river CH_4_ emissions [14]. Dedicated research is, however, needed to address the questions.

### Globally elevated CH_4_ emissions from nutrient-enriched rivers

The strong positive correlations between TP and NH_4_^+^-N and CH_4_ emissions suggest a linkage between anthropogenically enriched nutrient concentrations and elevated CH_4_ emissions from human-impacted rivers, especially since other aquatic variables did not significantly differ between human-impacted and pristine rivers (Fig. S6) or did not exhibit increasingly stronger impacts in human-impacted rivers (Fig. S13). This linkage bears important implications for both global river CH_4_ emissions and their potential mitigation, given that nutrient enrichment is mostly anthropogenically driven [46] and signifies a clear human footprint on river biogeochemistry and processes driving CH_4_ emissions [47]. Here, we identified all CH_4_ measurements that fall within human-impacted landscapes (urban settlements, croplands, or regions with population density >20 people km^−2^, Fig. 1a) and modelled them against both nutrient (TP, NH_4_^+^-N, TN and NO_3_^−^-N) concentrations that dictate the important *in situ* nutrient enrichment controls on CH_4_ emissions, and four types of terrestrial upland predictors (physical, climatic, pedologic and biological) previously used for estimating CH_4_ emissions from global rivers [5] (see Materials and Methods). The identified human-impacted regions make up only ∼28% of the Earth’s land area (excluding Antarctic), but host 96% of the world’s population, and therefore suggested to cover the majority of the world’s human-impacted nutrient-enriched rivers.

**Figure 2.**
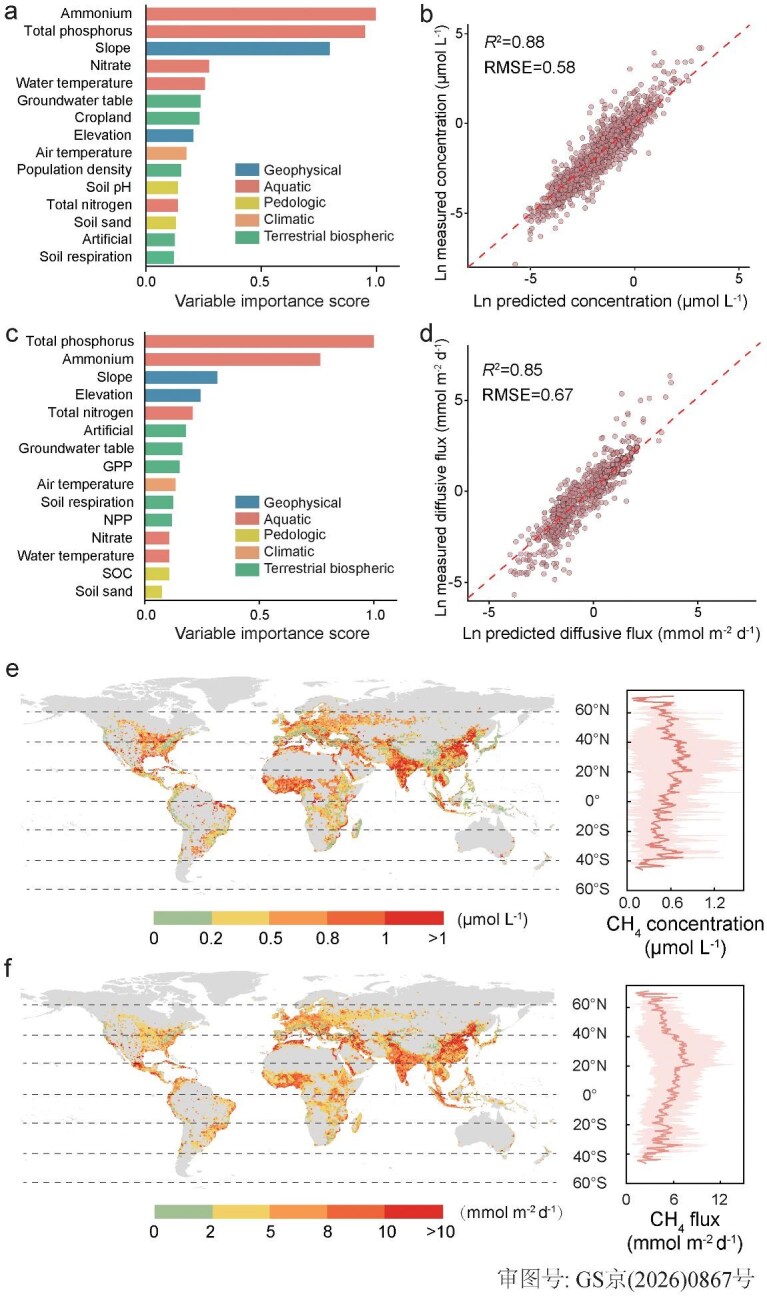
Variable importance, model performance, and predicted CH_4_ concentrations and fluxes of global human-impacted nutrient-enriched rivers. (a–d) Variable importance (top 15 predictors) and model performance of CH_4_ concentrations (a, b) and diffusive fluxes (c, d) after averaging the results of three ML methods. Dashed line represents the 1:1 line. *R*^2^ is the regression coefficient of the linear regression and RMSE is the root mean square error. (e, f) Spatial patterns of predicted CH_4_ concentrations and fluxes in human-impacted nutrient-enriched rivers and their latitudial variations. The dark red line and light red stripe of latitudial variation pannel represent the mean value and standard deviation of each latitude band, respectively. Variables include slope, elevation, total phosphorus, ammonium, nitrate, total nitrogen, water temperature, soil sand proportion (Soil sand), soil pH, soil organic carbon (SOC), population density, artificial land proportion (Artificial), cropland proportion (Cropland), gross primary production (GPP), net primary production (NPP), soil respiration, groundwater table, and air temperature. The base map in (e and f) is applied from the Database of Global Administrative Areas (https://gadm.org/).

Our analysis indicated that incorporating aquatic nutrient variables substantially improved model performance (Figs 2 and S14, Table S1), with TP and NH_4_^+^-N concentrations emerging as the most important predictors in the machine learning models (Fig. 2a, c). This aligned with the observation that CH_4_ concentration and flux were most strongly correlated with these two nutrient species in human-impacted rivers (Fig. 1). This underscores the importance of aquatic nutrient enrichment in driving variations in CH_4_ emissions from human-impacted rivers. Omitting their effects would likely lead to biased estimates of CH_4_ emissions from these rivers.

Predicted CH_4_ concentration and flux in human-impacted rivers ranged from 0.15 to 1.17 μmol L^−1^ (10th to 90th percentiles, same below) and from 2.5 to 10.6 mmol m^−2^ d^−1^, respectively (Fig. 2e, f). These were substantially higher than predicted for the same rivers from the previous estimate for global rivers (0.21–0.61 μmol L^−1^ and 0.91–2.89 mmol m^−2^ d^−1^) [5], suggesting probable underestimation without accounting for the effects of nutrient enrichment on CH_4_ emissions from human-impacted rivers. Importantly, in contrast to high CH_4_ emissions predicted for tropical (10°S–10°N) and high-latitude (>50°N) rivers in the previous study [5], the highest CH_4_ concentration and flux were observed in northern temperate rivers (20°–40°N) (Figs 2e, f and S15), which were associated concomitantly with the highest nutrient concentrations [20,48]. Particularly, rivers draining in eastern and southern Asia, western Africa, and mid-southern Europe exhibited higher CH_4_ emission rates compared to rivers draining the rest of human-impacted landscapes (Fig. 2e, f). These regions are among the most densely populated and suffer intense anthropogenic disturbances worldwide [49,50], suggesting a previously unrevealed linkage between human disturbances, aquatic nutrient enrichment, and elevated fluvial CH_4_ emissions.

### Global mitigation potential for CH_4_ emissions from nutrient-enriched rivers

**Figure 3.**
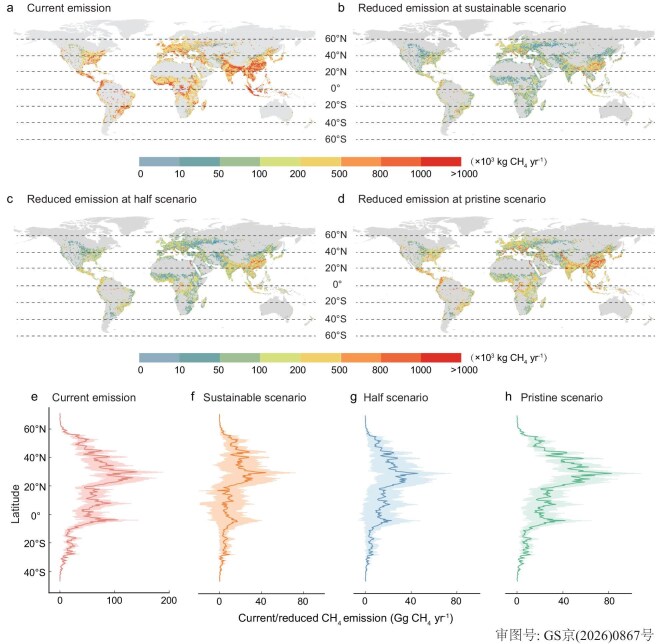
Global patterns of CH_4_ emissions and mitigation potential from nutrient-enriched rivers. (a–d) Geographical distribution of predicted CH_4_ emission from nutrient-enriched rivers and reduced emission under three scenarios at 0.5°×0.5° grid cell. (e–h) Latitudinal distribution of current CH_4_ emission (e) and reduced emissions under three scenarios (f–h). The base map in (a–d) is applied from the Database of Global Administrative Areas (https://gadm.org/).

Globally, we estimated CH_4_ emissions from human-impacted nutrient-enriched rivers at 9.7 ± 1.1 Tg CH_4_ yr⁻¹ after correcting for emissions during dry and ice-covered periods (see Materials and Methods; Fig. 3a). This represents 35% of global riverine emissions [5], yet exceeds prior estimates for the same rivers by 54% (6.3 Tg CH_4_ yr⁻¹) in the earlier study [5]. This marked increase likely results from human-induced nutrient enrichment (Fig. 1b–k) [47,51]. Here, utilizing predictive capacity of the machine learning models, we assessed how managing nutrient enrichment in human-impacted rivers can reduce the emissions under three hypothetical scenarios. The first is a “sustainable” scenario where nutrient (TP, NH_4_^+^-N, TN and NO_3_^−^-N) concentrations are reduced to meet key Sustainable Development Goals (SDGs) by 2030 (Fig. 3b). The second is a “half” scenario where nutrient concentrations are half of their current levels to align with the United Nations’ aspiration (Fig. 3c) and the third is a “pristine” scenario where nutrient concentrations are as in pristine rivers in geographically adjacent regions (Fig. 3d; see Materials and Methods). We assume the three scenarios represent a practical sustainable development pathway, a future condition of successful eutrophication control, and a theoretical benchmark for maximum mitigation potential from nutrient-enriched rivers, respectively.

**Figure 4.**
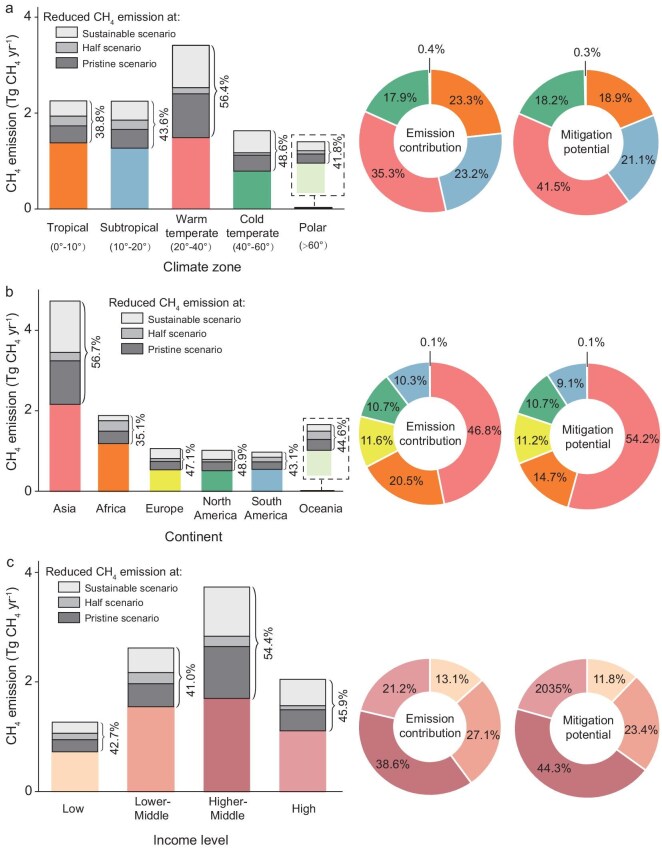
Global mitigation potential for CH_4_ emissions from nutrient-enriched rivers. The height of the entire bar indicates current CH_4_ emission from human-impacted rivers across climate zones (a), continents (b), and income levels (c). The height of the grey bar indicates the reduced CH_4_ emission from human-impacted rivers under three scenarios. Figures beside the bar represent the proportions of reduced CH_4_ emission under pristine scenario. Pie charts illustrate the contributions of each climate zone, continent, and income-level region to total CH_4_ emissions (left) and to the reduced CH_4_ emissions under the pristine scenario (right).

The analysis suggested a reduction in emissions of 2.2 ± 1.3, 2.7 ± 1.3, and 4.7 ± 1.2 Tg CH_4_ yr^−1^ under the three scenarios, respectively, accounting for 22%–49% of the estimated total of global nutrient-enriched rivers, indicating considerable mitigation potential through river nutrient control in nutrient-enriched rivers. Specifically, warm temperate rivers contributed the largest proportion to the mitigation (42%), though their share in the global total was only 35% (Fig. 4a). These rivers displayed the highest percentage of mitigation (26%–56% under the three scenarios), in line with the strongest nutrient enrichment in rivers in this region [52]. Among the major continents, Asian rivers contributed both the highest emissions (47%) and the highest percentage of mitigation (54%) (Fig. 4b), due to their largest share in global human-impacted rivers and strong nutrient enrichment in rivers in this region. Among the national income groups, the upper-middle-income countries shared both the largest emissions (39%) and percentage mitigation (44%) with 54% of the emissions being reducible under the pristine scenario (Fig. 4c), underscoring the substantial CH_4_ mitigation potential in rivers of upper-middle-income countries through implementation of cleaner technologies, stricter pollution controls, and enhanced water quality management [53].

With the current widespread agricultural intensification and urbanization, nutrient enrichment is expected to increase in freshwater ecosystems [54,55]. Our analysis reveals a strong linkage between CH_4_ emissions and aquatic nutrient enrichment in human-impacted rivers, suggesting these emissions are likely to rise significantly unless proactive measures are taken to prevent further nutrient enrichment. Given this linkage, mitigating anthropogenic nutrient enrichment represents a crucial strategy to curb riverine CH_4_ emissions, as supported by our evaluation of emission mitigation potential under various nutrient reduction scenarios.

Addressing nutrient pollution necessitates an integrated, multi-tiered approach encompassing watershed source control, riparian interception, and in-stream ecological restoration, with priority given to reducing nutrient inputs at the source. Agricultural croplands, as predominant nutrient sources, offer substantial mitigation potential [56]. For instance, the adoption of “Integrated Soil-Crop System Management” in China has demonstrated that optimized fertilizer application and enhanced Nitrogen Use Efficiency can reduce nitrogen leaching and runoff by up to 30% while simultaneously increasing crop yields [57]. In urban settings, upgrading wastewater treatment infrastructure with advanced nutrient-removal technologies can substantially reduce nitrogen and phosphorus loads. Evidence from wastewater-dominated rivers in Beijing indicates that such upgrades can reduce nutrient loads by over 50%, directly alleviating downstream eutrophication [58]. Beyond source control, enhancing the biogeochemical filter of river networks offers a highly cost‑effective complement. Restoring riparian buffer zones and vegetated filter strips can intercept 40%–90% of total nitrogen and phosphorus from surface runoff [59]. Similarly, establishing constructed wetlands at the interface of agricultural drains and streams can capture over 60% of nutrients, as evidenced by global meta-analyses [60]. Furthermore, in-stream restoration measures—such as sediment dredging, aeration, and phytoremediation—can play a pivotal role in nutrient reduction and water quality improvement [61,62]. A case study from the urban river network in Wuxi reported that comprehensive in-stream ecological restoration not only improved water quality but also substantially reduced CH_4_ fluxes [63]. Therefore, integrating watershed management, riparian interception, and in-stream restoration is essential to achieving synergistic co-benefits for water quality improvement and CH_4_ mitigation. Such efforts will accelerate progress toward nutrient-related SDGs and meaningfully contribute to carbon neutrality targets.

## MATERIALS AND METHODS

### Study area in China

We conducted field sampling campaigns in Beijing urban rivers and six large river networks in China, including the Pearl River, Yangtze River, Huai River, Hai River, Yellow River, and Liao River, from 2017 to 2022 (Fig. S1a). Beijing urban rivers flow through the capital city (39°56′ N, 116°20′ E), a megacity with 21 million inhabitants. The six large river networks (21°31′ to 45°10′ N, 90°13′ to 125°28′ E) primarily flow through the populated regions of eastern and southern China. A total of 138 reaches were investigated seasonally along the studied rivers, selected to encompass a wide gradient of anthropogenic influences, including population densities (70–33 175 people km^−2^) and land use types (i.e. urban settlements, cropland, forest, and grassland) of the adjacent watersheds. Due to strong human disturbance, these river reaches are enriched in nutrients (Fig. S2) and provide representative systems for understanding the impact of nutrient enrichment on CH_4_ emission at a broad scale. Detailed information about the sampling is described in the Supplementary Materials.

### GRiMeDB and additional global measurements

Global River Methane Database (GRiMeDB) [27], a comprehensive global dataset of riverine CH_4_ concentration and flux from multiple continents (Fig. S16), was utilized to explore the impacts of aquatic variables on CH_4_ emissions across diverse river systems worldwide. In addition to the data from GRiMeDB, we have updated the dataset with the latest measurements of riverine CH_4_ concentration and flux, as well as associated aquatic variables from 2022 to 2025, following the same data collection and screening protocols established by GRiMeDB (cut-off date on 30 September 2025). For each sampling site, we averaged the measurements from multiple grab samples collected over time. Furthermore, we have integrated our field measurements from Chinese rivers to the dataset. These updates collectively contribute 3303 new measurements from 53 publications, strengthening the comprehensiveness and reliability of the global riverine CH_4_ database. The updated dataset now contains 25 839 measurements of CH_4_ concentrations, 6239 measurements of diffusive CH_4_ flux measurements, 6239 measurements of TP, 10 340 measurements of NH_4_^+^-N, 7649 measurements of TN, 13 289 measurements of NO_3_^−^-N, 17 316 measurements of DOC, 22 812 measurements of water temperature, 10 656 measurements of DO, 15 735 measurements of pH, and spread over a wide range of ecosystems.

### Predicting CH_4_ concentrations and fluxes of human-impacted rivers

We employed three machine learning algorithms [i.e. random forest, XGBoost, and support vector machine (SVM)] to predict CH_4_ concentrations and diffusive fluxes of global human-impacted rivers (see Supplementary Materials for identification of human-impacted rivers). The modeling framework incorporated reach-scale environmental predictors to model against collated CH_4_ concentrations and diffusive fluxes of human-impacted rivers. Reach-scale predictors including terrestrial upland variables (geophysical, climatic, edaphic, and terrestrial biospheric attributes) and in-stream controls (aquatic variables) were initially filtered through correlation analysis (*r* > 0.1, Fig. S17). Terrestrial variables with weak but mechanistically relevant correlations (e.g. precipitation, gross primary productivity, net primary productivity, and soil respiration) were also selected given their established roles in regulating riverine CH_4_ dynamics. Finally, 21 reach-scale variables (16 terrestrial variables and 5 aquatic variables) were selected for modeling (Fig. S17). Five aquatic variables included four nutrient concentrations (TP, NH_4_^+^-N, TN, NO_3_^−^-N) and water temperature. Among the 21 predictor variables, air temperature, water temperature, precipitation, gross primary productivity, net primary productivity, soil respiration, and groundwater table had monthly values that were aligned with the CH_4_ concentrations and diffusive fluxes for the corresponding month in the global CH_4_ dataset. Prior to model development, all strongly skewed predictors underwent logarithmic transformation to meet normality assumption. All of those predictors were spatially matched to the GRADES reaches (see Supplementary Materials).

Modeling was performed by matching CH_4_ concentrations and diffusive fluxes with the reach-scale predictors using randomForest, xgboost, and e1071 packages in R (v.4.0.3) for random forest, XGBoost, and SVM modeling, respectively. Before modeling, we performed systematic hyperparameter tuning to identify the optimal model configuration (Table S2). Tuning was conducted by constructing a hyperparameter grid that encompassed broad ranges of the parameters. The modeling was repeated with a random combination of the three model parameters by enumeration. The best parameter combination was determined via 10-fold cross-validation when the mean of squared residuals was minimized, with the optimal values presented in Table S2. A Leave-One-Out Cross Validation method was used to evaluate the model robustness. In this process, the model was iteratively trained on n−1 data points (where n represents the total number of observations), with each iteration reserving a single distinct observation for validation. Model performance was evaluated by comparing predicted values against corresponding measured values across all iterations. All three machine learning algorithms demonstrated robust predictive performance for both CH_4_ concentration (*R*^2^ = 0.70–0.77) and diffusive flux (*R*^2^ = 0.67–0.71, Table S1). We therefore employed ensemble averaging of their predictions to produce final estimates (Fig. 2), enhancing model stability and reducing algorithm-specific biases. The reach-scale predictors were ranked based on their importance using different metrics: increase in mean squared error (%IncMSE) for Random Forest, Shapley Additive Explanations (SHAP) values for XGBoost, and permutation importance for SVM. These algorithm-specific importance metrics were first normalized to a 0–1 scale, then averaged across all three algorithms to produce robust, ensemble-based variable importance rankings. The top 15 predictors were identified according to their averaged importance rankings (Fig. 2a–d). To evaluate the importance of aquatic nutrient variables (TP, NH_4_^+^-N, TN, NO_3_^−^-N), we compared the performance of the models by including and excluding nutrient variables from the reach-scale predictors, respectively. A sensitivity analysis was further performed by replacing the four most influential watershed variables with nutrient variables (TN, TP, NH_4_^+^, NO_3_^−^), thereby eliminating potential interference from simply adding more predictors (Table S3). Partial dependence plots were created using *rpart* package (v.4.1-15) in R to examine the marginal effect of each variable on the modelled CH_4_ concentrations (Fig. S18).

To capture the seasonal patterns, monthly models were constructed using the observations from a given month and its adjacent months (e.g. January model was constructed using observations from December, January, and February) to increase the number of observations. For each monthly model, 70% of the CH_4_ concentrations and diffusive fluxes were randomly chosen for training and the remaining for testing. The monthly models had good predictive performance for Ln CH_4_ concentrations (*R*^2^ = 0.65–0.88) and Ln diffusive fluxes (*R*^2^ = 0.56–0.75, Fig. S19). To account for the influence of nutrient levels on the diffusive-to-ebullitive flux ratio, we established dynamic relationships stratified by population density groups (Fig. S20), a robust proxy for anthropogenic nutrient loading. Ebullitive flux for each river segment was then estimated from diffusive flux using the relationship specific to its population density group.

### Upscaling CH_4_ emissions and mitigation potential from human-impacted rivers

We predicted CH_4_ concentrations and fluxes generated from monthly models through three machine learning algorithms. Before model prediction, all reach-scale predictors were spatially aggregated at 0.5°×0.5° grid cells based on the geographical position of river reaches and grid cell boundaries. The mean nutrient concentrations were calculated as the total nutrient loads divided by the cumulative runoff in each grid cell. The global nutrient loads in rivers at 0.5°×0.5° grid cells were sourced from the Integrated Model to Assess the Global Environment–Global Nutrient Model (IMAGE-GNM) [52,64] (see Supplementary Materials). We estimated monthly CH_4_ emissions for 0.5°×0.5° grid cells by multiplying CH_4_ fluxes with the corresponding monthly river surface area within each grid cell. The emissions for each month were then accumulated to calculate the annual CH_4_ emissions. The monthly river surface area was calculated based on the GRADES river networks as the product of the monthly width and reach length. For the smallest rivers not captured by GRADES networks, we extrapolated the river area using scaling relationships with Strahler stream order following methods from Liu *et al.* [65]. The extrapolated area was also aggregated within a 0.5°×0.5° grid cell on a monthly scale. The monthly river surface area was corrected for periods when rivers were dry or covered by ice [65], under the assumption that CH_4_ evasion was prevented during these conditions [5].

We set three nutrient reduction scenarios, the sustainable scenario, half scenario, and pristine scenario, to assess the CH_4_ mitigation potential from human-impacted rivers. Sustainable scenario refers to the water quality conditions necessary for achieving key SDGs by 2030 [56]. Nitrogen concentrations are derived by applying the country-specific reduction ratio relative to current levels (Table S4), which is required to meet balanced nitrogen-related SDG targets. As a co-limiting nutrient, an equivalent reduction ratio for phosphorus is assumed to reflect synergistic management. Half scenario refers to the nutrient concentrations being half of the current state in human-impacted rivers, given that global N and P loads to river networks and their concentrations have more than doubled over the past century (Fig. S21) [64]. This scenario also aligns with the ambitious United Nations aspiration to halve nitrogen and phosphorus waste by 2030 to reduce pollution risks [66,67]. Pristine scenario refers to the nutrient concentrations decreasing to natural or seminatural levels (i.e. nutrient concentrations of pristine rivers within the same basin of HydroBASINS Level 2), representing the maximum emission mitigation potential from human-impacted rivers. Pristine scenario also signifies the theoretically increased riverine CH_4_ emissions attributable to human-driven nutrient enrichment since the industrial era. We estimated CH_4_ emissions from human-impacted rivers under three nutrient reduction scenarios through the same machine learning models using the corresponding nutrient levels and other predictors. CH_4_ emission mitigation potential from human-impacted rivers was calculated as the differences between current CH_4_ emissions and those under three nutrient reduction scenarios.

### Statistical analysis

Statistical analyses were carried out in R (version 4.0.3), Microsoft Excel (version 2019), and OriginPro (version 2024). Before analysis, the normality of all variables was assessed using the Shapiro-Wilk test. Variables with non-normal distributions were logarithmically transformed to approximate normal distributions. Standardized linear regressions were utilized to explore the impacts of aquatic and terrestrial variables on CH_4_ emissions from rivers. Prior to analysis, all variables were standardized by converting them into Z-scores. The absolute values of the regression coefficients were then used to compare the correlation strength of the variables after performing the regression analysis. Significant differences in CH_4_ and aquatic variables across different groups were tested using the Wilcoxon rank-sum test.

## Supplementary Material

nwag192_Supplemental_File

## Data Availability

All data including CH_4_ raw data in Chinese rivers, the updated GRiMeDB, and the codes for upscaling global CH_4_ emissions and uncertainty analysis are available in the Figshare repository (https://doi.org/10.6084/m9.figshare.29364617).
