# Supplementary material for: Globally doubled methane emissions from nutrient-enriched rivers
Source: Natl Sci Rev. 2026 Mar 26;13(8):nwag192. doi: 10.1093/nsr/nwag192 (PMC13170804; doi:10.1093/nsr/nwag192)
Supplement: nwag192_Supplemental_File [file nwag192_supplemental_file.docx]

Supplementary materials for

Globally doubled methane emissions from nutrient-enriched rivers

Junfeng Wang, Xinghui Xia, Shaoda Liu, Sibo Zhang, Junyu Dong, Gongqin Wang, Wenhao Xu, Ling Zhang, Wenxiu Zheng, Zhuangzhuang Zhang, Xin Chen, Linfeng Yuan, Jiao Liu, Jiajia Zhang, Yuan Xin, William H. McDowell, Hanqin Tian, David Bastviken, and Zhifeng Yang

Corresponding author: Xinghui Xia, Shaoda Liu

Email: xiaxh@bnu.edu.cn; liushaoda@bnu.edu.cn

**This file includes**:

Supporting methods

Figs. S1 to S26

Tables S1 to S5

References

# Supporting Methods

## Field sampling of Chinese rivers

This study conducted the field sampling surveys in Beijing urban rivers and six large river networks in China from 2017–2022 (Fig. S1a). For Beijing urban rivers, a total of 19 sites were sampled in 18 sampling campaigns from July 2018 to September 2020. Biweekly observations were conducted at 8 river sites from July 2018 to July 2019 (except frozen periods between December and February), and all 19 sites were sampled in August 2019, November 2019, and September 2020. In total, 196 observations were obtained from the 18 sampling campaigns. For six river networks, field sampling was conducted at 119 river reaches across different land use types and population density gradients during a consecutive 6-year period from 2017 to 2022. Two or three river networks were investigated every year. Three seasonal sampling events, including spring (March or April), summer (August), and autumn-winter (October, November, or December), were performed in each river network from 2017–2021. In addition, we performed investigations with high spatial resolution at 35 sampling sites of the Hai River in August, 2022. In total, 287 observations were obtained from the sampling campaigns in six river networks.

## Field sampling and laboratory analysis

At each sampling site, we conducted *in situ* measurements and collected samples to determine the dissolved CH_4_ concentrations, CH_4_ fluxes, and riverine physicochemical and hydrological characteristics. Surface water samples (~10 cm below the surface) were collected with acid-washed polypropylene bottles for the determination of dissolved organic carbon (DOC), dissolved inorganic nitrogen (i.e., NH_4_^+^-N and NO_3_^-^-N), total nitrogen (TN), and total phosphorus (TP). Water samples were filtered *in situ* with 0.45-μm membrane filters (Supor 450, Pall Corporation, USA) and acidified with H_2_SO_4_ to pH < 2 for preservation. In addition, 0.7-μm glass microfiber filters (GF/F 1825-047, Whatman, UK) were used to filter certain amounts of surface water and stored in dark for determination of the concentration of chlorophyll *a*. All samples were stored at 4 °C in a portable refrigerator until further analysis in the laboratory. During the 2021 field campaign, surface sediment samples (0–10 cm depth) were collected from 29 reaches along the Yellow River for subsequent molecular biological analysis. All samples were snap-frozen in the field and subsequently stored in liquid nitrogen. Water temperature, pH, dissolved oxygen (DO), and electrical conductivity were measured *in situ* using a portable multiparameter meter (HQ 40d, HACH, USA). Ambient air temperature, atmospheric pressure, and wind speed were measured with a portable anemometer (Testo 480, Germany). River discharge, flow velocity, channel width, and mean depth were obtained from local hydrological stations.

In the laboratory, filtered water samples were analyzed using high-temperature catalytic oxidation with a Shimadzu TOC-L analyzer (Shimadzu Scientific Instruments, Japan) to measure DOC concentrations. A colorimetric method was applied to determine the concentrations of NH_4_^+^-N, NO_3_^-^-N, and TN with an Autoanalyzer (A3, Seal, Germany). Concentrations of TP were determined with an ultraviolet-visible spectrophotometer (HACH, DR6000) after persulfate digestion using the colorimetric molybdenum blue method. Extracted from the glass microfiber filters with acetone, chlorophyll *a* concentrations were determined with an ultraviolet-visible spectrophotometer (HACH, DR6000) across a range of wavelengths (630–750 nm).

## CH_4_ concentration and flux measurements

Surface water samples were collected with a stainless-steel water sampler (Purity WB-SS, China) and then transferred to 120-ml glass serum vials via a flexible silicone tube for the determination of dissolved CH_4_ concentrations. The water samples were recollected if any bubbles appeared in the vials. The headspace equilibrium technique was adopted *in situ* to extract dissolved CH_4_ [[2](#_ENREF_2)]. Briefly, 10 ml ultrahigh-purity helium was injected into the glass vials, and an equivalent volume of water was replaced to create a headspace. Then, the glass vials were shaken vigorously to achieve gas equilibrium. After static equilibrium for 10 minutes, the equilibrated gas in the headspace was transferred into a pre-evacuated 30-ml aluminum foil airbag through a gastight syringe. At each sampling site, three floating chambers were deployed at the water surface to determine CH_4_ fluxes across water‒air interface. During sampling, 100 ml of homogeneous gas was collected through a polyurethane tube after thoroughly mixing the headspace gas in the chamber, then immediately transferred to pre-evacuated airtight bags. We collected gas samples in the chambers at 10-min intervals for a total duration of 60 min. The gas samples were stored in the dark and measured by a gas chromatograph equipped with a flame ionization detector (Agilent 7890B GC-FID, USA) in the laboratory.

Dissolved CH_4_ concentrations in water samples were calculated according to CH_4_ concentrations in the headspace and Bunsen solubility coefficients [[3](#_ENREF_3)]:

$C_{w}=\frac{C_{g}}{V_{w}}\times\left( \frac{\beta RTV_{w}}{V_{g}}+V_{g} \right)$ (S1)

where $C_{w}$ is the dissolved CH_4_ concentration in surface water (mol L^-1^), $C_{g}$ is the CH_4_ concentration in the headspace (mol L^-1^), $V_{w}$ is the volume of remaining water (L), $V_{g}$ is the volume of the headspace (L), $R$ is the gas constant (0.0821 atm·L·mol^-1^·K^-1^), $T$ is the air temperature (K) when equilibrating, $V_{g}$ is the ideal gas volume (L mol^-1^), $\beta$ is the Bunsen solubility coefficient (L·L^-1^·atm^-1^) which is calculated according to the following equation [[4](#_ENREF_4)]:

$ln\beta=A_{1}+A_{2}\left( \frac{100}{T} \right)+A_{3}In\left( \frac{T}{100} \right)+S\left[ B_{1}+B_{2}\left( \frac{T}{100} \right)+B_{3}\left( \frac{T}{100} \right)^{2} \right]$ (S2)

here $T$ is the water temperature (K), $S$ is the salinity (‰), $A_{1}$ to $B_{3}$ are constants.

At each sampling site, three floating chambers were deployed at the water surface to determine the CH_4_ and CO_2_ fluxes across the water-air interface. To minimize the heating effect on headspace gas, the chambers were covered with aluminium foil to reflect sunlight. Three duplicate samples were collected from each chamber at each time point. An ambient sample of atmospheric air was sampled simultaneously at each site. The CH_4_ and CO_2_ flux across the water-air interface was calculated according to the following equation:

$F_{T}=\frac{(C_{t}-C_{0})\times V_{c}}{A\times t}\times{10}^{-3}\times60\times24$ (S3)where $F_{T}$ (mmol m^-2^ d^-1^) is the CH_4_ and CO_2_ flux across the water-air interface; $C_{0}$ and $C_{t}$ (μnmol L^-1^) are the CH_4_ and CO_2_ concentrations in the chamber at time zero and time *t*, respectively; $V_{c}$ (L) is the chamber volume; *A* (m^2^) is the area of water surface covered by the chamber; and *t* (min) is the sampling time during which the CH_4_ and CO_2_ concentration in the chamber increases linearly to eliminate the possible biases due to gas accumulation.

Ebullition is an important pathway of CH_4_ evasion in river systems. To separate the CH_4_ transport pathways, we partitioned the measured water-air fluxes into diffusive and ebullitive fluxes based on the previous established method [[5](#_ENREF_5)]. The release of CO_2_ across the water-air interface is a strictly diffusive process, so the measured CO_2_ flux is exclusively attributed to diffusive flux which can also be calculated by Fick’s law of gas diffusion. The gas transfer velocity is determined by inverting Fick’s law as follows:

$k_{{CO}_{2}}=\frac{F_{{CO}_{2}}}{{CO}_{2water}-{CO}_{2eq}}$ (S4)

where $k_{{CO}_{2}}$ (m d^-1^) is the gas transfer velocity of CO_2_; $F_{{CO}_{2}}$ (mmol m^-2^ d^-1^) is the CO_2_ flux across water-air interface; ${CO}_{2water}$ and ${CO}_{2eq}$ (μmol L^-1^) are the dissolved CO_2_ concentration in surface water and the theoretical CO_2_ concentration in equilibrium with ambient air corrected for local water temperature and atmospheric pressure, respectively. Then the gas transfer velocity for CH_4_ diffusion was calculated based on $k_{{CO}_{2}}$ as follows:

$k_{{CH}_{4}}=k_{{CO}_{2}}\times\left( \frac{Sc_{{CH}_{4}}}{Sc_{{CO}_{2}}} \right)^{-n}$ (S5)

where $k_{{CH}_{4}}$ (m d^-1^) is the gas transfer velocity of CH_4_; $Sc_{{CH}_{4}}$ and $Sc_{{CO}_{2}}$ are Schmidt numbers as a function of temperature for CH_4_ and CO_2_, respectively; $n$ is Schmidt coefficient, which is equal to 2/3 and 1/2 for wind speeds < 3.6 m s^−1^ and > 3.6 m s^−1^ at 10 m height, respectively. $Sc_{{CH}_{4}}$ and $Sc_{{CO}_{2}}$ are calculated based on the following equations [[3](#_ENREF_3)]:

$Sc_{{CH}_{4}}=1897.8-114.28T+3.2902T^{2}-0.039061T^{3}$ (S6)

$Sc_{{CO}_{2}}=1911.1-118.11T+3.4527T^{2}-0.041320T^{3}$ (S7)

The conversion from $k_{{CO}_{2}}$ ​​to $k_{{CH}_{4}}$ is based on the relationship expressed in Equation S5, which assumes that gas transfer velocities can be interconverted via their Schmidt numbers. However, practical applications have shown discrepancies between the *k_600_*​ values derived from CO_2_ and CH_4_, primarily attributed to microbubble-mediated transport of CH_4_ and the chemical enhancement of CO_2_ exchange within the surface microlayer [[6](#_ENREF_6)]. These processes are known to be significant in high-energy environments, specifically in steep streams (slope > 0.06 m/m) or under high wind speeds (> 3 m/s) [[7](#_ENREF_7)]. In our study, over 90% of the sampling sites were characterized by gentle slopes (< 0.06 m/m) and low wind speeds (< 3 m/s) during measurement (Fig. S22), conditions that minimize the potential contribution of microbubble fluxes. Regarding chemical enhancement, we followed the method of [Kuss and Schneider [8]](#_ENREF_8) to calculate chemical enhancement factors (α) to further correct the *k*_600CO2_​​​ values:

$\alpha=1.6\times{10}^{-8}\times e^{1.98pH}+1$ (S8)

Although temperature-dependent chemical and biological CO_2_ production has a potential impact on $k_{{CO}_{2}}$, quantifying the temperature effect precisely is beyond the capacity of our current study. We highlight this as an important focus for future research.

The diffusive CH_4_ flux ($F_{d}$, mmol m^-2^ d^-1^) is calculated following the Fick’s law, and the difference between total and diffusive CH_4_ flux is attributed to ebullitive flux ($F_{e}$, mmol m^-2^ d^-1^):

$F_{d}=k_{{CH}_{4}}\times\left( {CH}_{4water}-{CH}_{4eq} \right)$ (S9)

$F_{e}=F_{T}-F_{d}$ (S10)

## Molecular biological analysis

Genomic DNA was extracted in triplicate from fresh sediment using the FastDNA SPIN Kit (MP Biomedicals, USA) according to the manufacturer's instructions. The quality and concentration of the extracted DNA were verified using a NanoDrop 2000 spectrophotometer (Thermo Scientific, USA) and a Qubit 4.0 Fluorometer (Invitrogen, USA). The abundance of the functional genes *mcrA* and *pmoA* in sediment samples was quantified via real-time quantitative PCR (qPCR) on an ABI7300 system (Applied Biosystems, USA). For *mcrA* gene amplification, the primer pair MLfF (5′-GGTGGTGTMGGATTCACACARTAYGCWACAGC-3′) and MLrR (5′-TTCATTGCRTAGTTWGGRTAGTT-3′) was used. The *pmoA* gene was amplified with primers A189F (5′-GGNGACTGGGACTTCTGG-3′) and mb661R (5′-CCGGMGCAACGTCYTTACC-3′). Quantification standard curves were constructed using ten-fold serial dilutions of plasmid DNA containing the respective target gene fragment. All qPCR assays met quality control criteria, with standard curve correlation coefficients (R^2^) above 0.99 and amplification efficiencies ranging from 90% to 110%.

For the analysis of methanogenic and methanotrophic communities of sediment samples, high-throughput sequencing was performed using the same primers *mcrA* and *pmoA*. The PCR products were purified with a GeneJET Gel Extraction Kit (Thermo Fisher Scientific, Waltham, MA, USA) and the amplicon sequencing libraries were generated on the Illumina MiSeq platform (MajorBio Company in Shanghai, China). Raw sequencing reads were processed using the QIIME 1.9.1 pipeline for quality control. Sequences were trimmed and filtered to remove low-quality reads (Phred score < Q20), short reads (< 150 bp), and those containing ambiguous bases with Trimmomatic (v0.39). Chimeric sequences were identified and removed using the DADA2 algorithm, which discriminates chimeras based on sequence abundance and alignment mismatches. After quality filtering, non-chimeric sequences were used for downstream analysis. The α-diversity of methanogenic and methanotrophic communities, including the Chao1 richness index, was calculated using the QIIME v1.9 pipeline to assess microbial diversity within each sediment sample.

## Global hydrographic frameworks

In this study, riverine CH_4_ concentration and flux need to be modeled against reach-scale environmental variables at the same temporal and spatial resolution, multiplying the corresponding river surface area to estimate CH_4_ emissions from global rivers. The *Global Reach-level A priori Discharge Estimates for SWOT* (GRADES) river networks [[9](#_ENREF_9)] (<http://hydrology.princeton.edu/data/mpan/MERIT_Basins/>), a new representation of global river networks derived from the fine resolution (~ 90 meters) *Multiple-Erro-Removed Improved-Terrain Digital Elevation Model* (MERIT DEM) and related hydrography datasets [[10](#_ENREF_10)], were used as the underlying hydrographic infrastructure for the riverine CH_4_ estimates. GRADES contains daily discharge estimates for all river reaches over a 35-year period (1979–2014).

We calculated each of the ~ 3 million GRADES river reaches for reach-level flow velocity (*V*) through discharge (*Q*) based on the topological relationships between them. The equitation presented by Raymond et al. (i.e., ln *V* = 0.12 ln *Q* – 1.06) [[11](#_ENREF_11)], which was derived from 9,811 US geological survey (USGS) gauging stations having > 20 instantaneous hydraulics measurements, was chosen to scale *V* across GRADES river networks. This equitation was also used to estimate *V* in a previous study for upscaling CO_2_ emissions from global rivers, and was proved to yield reliable *V* estimates for both the local and national river networks [[12](#_ENREF_12)]. The channel slope was determined using stream lines from HydroSHEDS and elevation from USGS Global Multi-resolution terrain elevation data [[13](#_ENREF_13)]. The surface area of the ~ 3 million GRADES river reaches was estimated using the length of each river reach multiplied by the corresponding width. Monthly widths of a reach were estimated by coupling downstream and at-a-station hydraulic geometries with reach-level monthly discharge estimates from GRADES [[12](#_ENREF_12)]. The smallest river reaches not represented by the GRADES was provided by ref. 12 and estimated by extrapolating existing reach-level GRADES surface area using scaling relationships with Strahler stream order. The extrapolated area was resolved at a HydroBASIN 04 level and at a monthly scale, which was downscaled to each 0.5°×0.5° grid cell for use in emissions estimates.

## Extents of human-impacted regions

Urban settlements and croplands are categorized as human-impacted regions in this study, given their susceptibility to intensive anthropogenic pressures such as urban development, resource extraction, intensive farming practices, and various forms of pollution [[14](#_ENREF_14)]. In addition, densely populated areas exceeding a defined population density threshold are also identified as human-impacted regions. To determine the population density threshold, we performed a moving subset window analysis to assess significant variations in the dissolved concentration of four nutrients (TP, NH_4_^+^-N, TN, NO_3_^-^-N) across different population densities (Fig. S23), given that their widespread positive correlations with population density globally [[15](#_ENREF_15),[16](#_ENREF_16)]. The analysis employed adaptive window parameters, using 10-unit and 100-unit windows for population density below and above 100 people km^-2^, respectively, with corresponding step increments scaled with population density ranges (1 unit for 0-10, 10 for 10-100, and 100 for 100-1000 people km^-2^). The upper limit of a population density subset was identified as the threshold, where its nutrient concentrations significantly exceeded those of the preceding subset, while no significant differences were observed among the following three subsets. Through this approach, we identified the population density thresholds as 11–14 people km^-2^ across four nutrient analyses (Fig. S23). Conservatively, a population density threshold of 20 people km^-2^ was implemented to delineate human-impacted regions. The selected threshold is supported by the stabilization of strong CH_4_-nutrient correlations when the analysis was constrained to areas with population density > 20 people km^-2^ (Fig. S24), as evidenced by the flattening regression slopes in Fig. 1k, supporting the appropriateness of our selected threshold for isolating a clear human-impact signal. We therefore defined human-impacted regions as the combination of urban settlements, croplands, and densely populated (> 20 people km^-2^) regions. These defined human-impacted regions also successfully capture areas of high anthropogenic intensity characterized by Human Footprint Index (HFI) and Nighttime Light intensity (NTL), two widely used, globally-consistent indices reflecting human disturbance degrees (Fig. S25). Based on this definition, we determined the extents and geographical distribution of human-impacted regions (Fig. 1a). Human-impacted regions constitute 28% of the total continental area (Antarctic excluded) but accommodate 96% of the world's population, which mainly include central Africa, India, eastern and southern Asia, Europe, and eastern North America (Fig. 1a). Urban boundaries were delineated based on a vector database of Global Hierarchical Urban Boundaries (GHUBs) [[17](#_ENREF_17)]. Cropland areas were defined using a 250-m resolution global land cover product [[18](#_ENREF_18)] (available at <https://data-starcloud.pcl.ac.cn/iearthdata/>). World population density (people km^-2^, at ~ 1 km resolution) is obtained from Socioeconomic Data and Applications Center, which can be accessed via  <https://doi.org/10.7927/H4NP22DQ>.

To determine river networks impacted by human disturbance, we overlapped the extents of global human-impacted regions and GRADES [[9](#_ENREF_9)], which contains around 3 million river reaches. River reaches from the GRADES database within the boundaries of global human-impacted regions were defined as human-impacted rivers, while those outside this boundary were considered undisturbed rivers. Totally, ~ 0.9 million river reaches were selected as human-impacted reaches from GRADES, which accounts for ~34% of total GRADES area.

## Nutrient load and concentration

We obtained global riverine total phosphorus (TP) and total nitrogen (TN) loads at 0.5° resolution from the *Integrated Model to Assess the Global Environment–Global Nutrient Model* (IMAGE-GNM) [[19](#_ENREF_19),[20](#_ENREF_20)]. The IMAGE-GNM is a global, spatially explicit, distributed model that simulates TN and TP flows entering river networks worldwide at an annual time step from multiple sources, including atmospheric deposition, aquaculture, wastewater, runoff, groundwater, vegetation in floodplains and chemical weathering [[20](#_ENREF_20)]. We calculated total phosphorus and nitrogen loads by aggregating inputs from all sources. To estimate NH_4_^+^ and NO_3_^-^ loads, we first determined their fractional contributions to TN for each source based on regional and global-scale studies (Table S5). We then derived NH_4_^+^ and NO_3_^-^ loads by applying these source-specific proportions to the corresponding TN loads. Total nutrient load in the channel of a cell was defined as the sum of the outflow of all upstream cells plus the local loads. The mean nutrient concentration of each cell was calculated as the ratio of nutrient loads to the cumulative runoff.

## Uncertainty analysis

We evaluated two main sources of uncertainty in estimating riverine CH_4_ emissions: river surface area and the modeled CH_4_ fluxes. Given that river surface area were calculated by river reach length and width, which were estimated by discharge [[12](#_ENREF_12)], we used the uncertainty in river discharge for each reach from GRADES. The residual error in discharge was fitted to a normal distribution by comparing the discharge of GRADES with the discharge data from USGS stations. The mean error and one standard deviation were determined to be -0.02 and 1.22 for ln discharge, generating a normal distribution of error with a mean error of -0.008 and a standard deviation of 0.52 for ln width (ln width = 2.56 + 0.423 × ln discharge) [[21](#_ENREF_21)]. Then, the uncertainties in river surface area were quantified based on their relationships with width through error propagation. The uncertainty induced by the predictive model was evaluated by fitting the model residuals to a log-normal distribution. The error at one standard deviation was then determined for both ln CH_4_ concentration and ln CH_4_ diffusive flux (Fig. S26). To evaluate the uncertainty of riverine CH_4_ emissions, we performed Monte Carlo simulation by generating random values from normal distributions of each uncertainty source defined above for 1000 times. These uncertainties were then propagated to CH_4_ emission estimates through the prediction processes. The final uncertainty was determined based on the distribution of CH_4_ emission from Monte Carlo simulation and were reported as the mean ± 1 standard error of the emissions. Monte Carol analysis was conducted in R (v.4.0.3) to evaluate the uncertainty.

# Supporting Figures


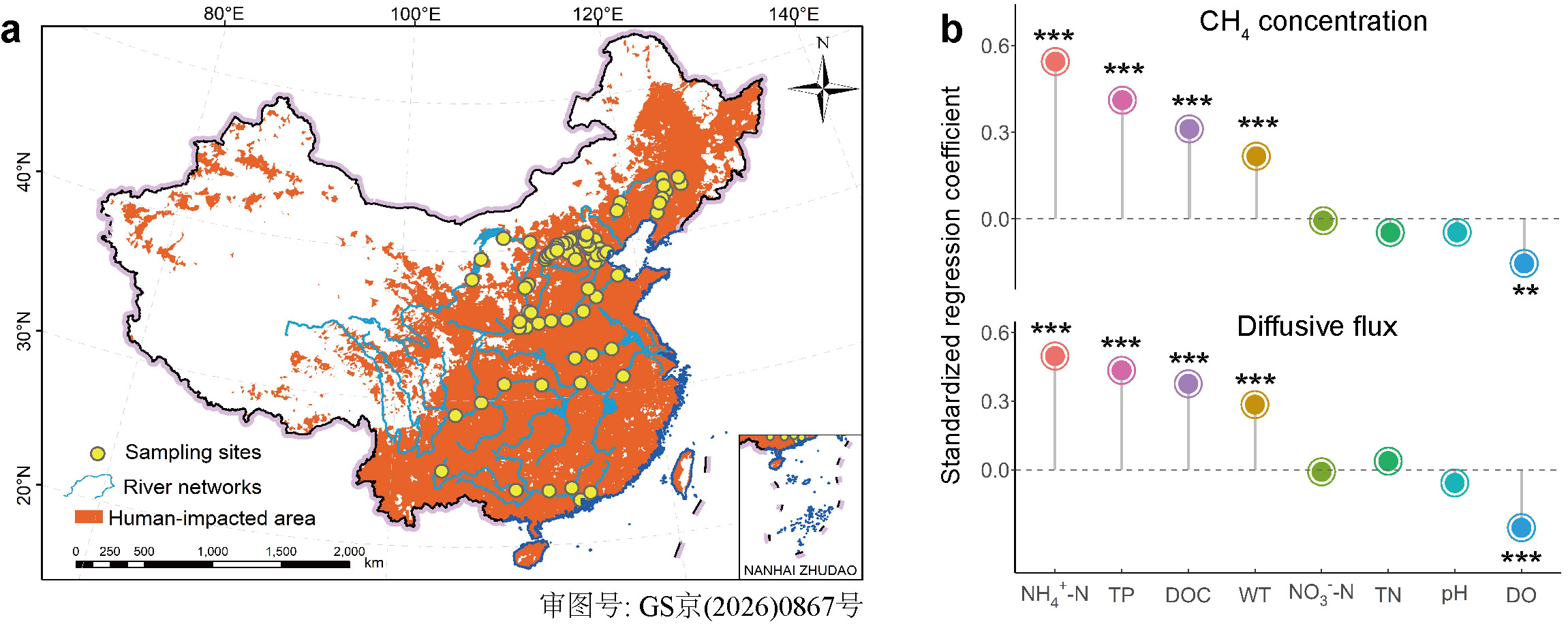
**Figure S1 Relationships between CH_4_ concentration and diffusive flux and eight aquatic variables across Chinese dataset. a** Geographical distributions of sampling sites of Chinese rivers and the extent of human-impacted area. The zoomed-in map displays the sampling sites of Beijing urban rivers. **b** Standardized regression coefficients between CH_4_ concentration and fluxes and eight aquatic variables across Chinese dataset. Human-impacted rivers refer to rivers situated in areas impacted by human activities, including urban settlements, croplands, and densely populated regions (i.e. > 20 people km^-2^). All variables were standardized using the Z-score normalization method before standardized linear regressions. The eight aquatic variables include: NH_4_^+^-N, ammonium nitrogen; TP, total phosphorus; DOC, dissolved organic carbon; WT, water temperature; NO_3_^-^-N, nitrate nitrogen; TN, total nitrogen; pH, pH value; DO, dissolved oxygen. The asterisks above the sticks indicate the statistical significance of the regression results. Significance levels are represented as follows: ***, *p* < 0.001; **, *p* < 0.01; *, *p* < 0.05. The base map of China was obtained from the standard map service of the Ministry of Natural Resources (http://bzdt.ch.mnr.gov.cn/).


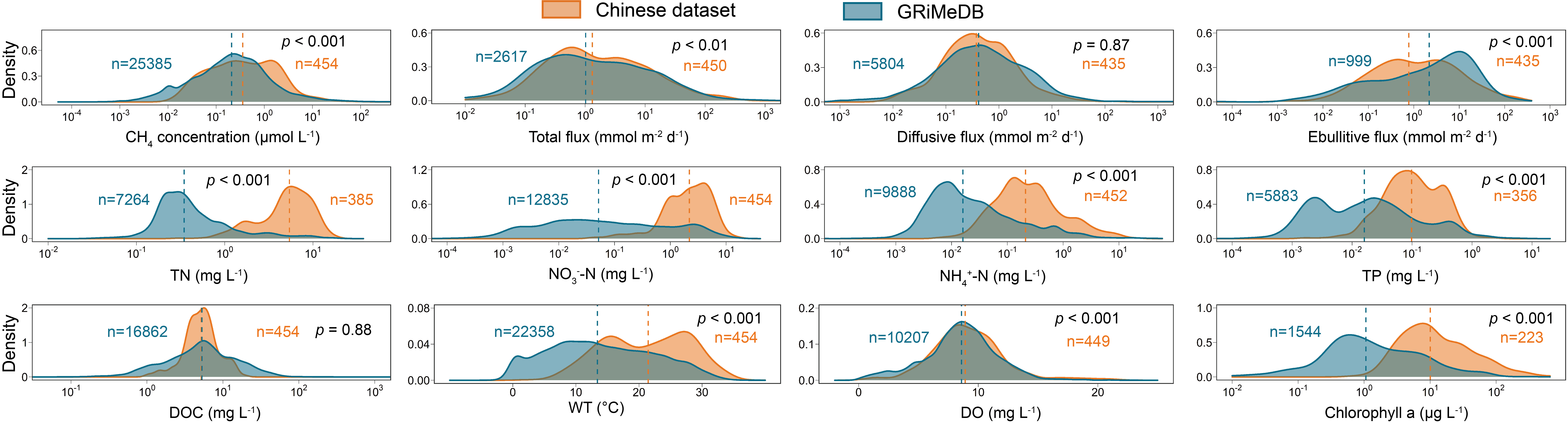
**Figure S2 Distribution density curves of CH_4_ concentrations and fluxes and** **associated aquatic variables across Chinese dataset and the updated GRiMeDB.** Chinese dataset includes 483 measurements from the populated middle and eastern Chinese rivers. GRiMeDB [[22](#_ENREF_22)] is updated with the latest measurements of riverine CH_4_ characteristics and associated aquatic variables (see Methods). The dashed line represents the median value of each variable across the datasets. The *p* values denote the statistical significance between the two groups, which was tested with Wilcoxon rank-sum test.


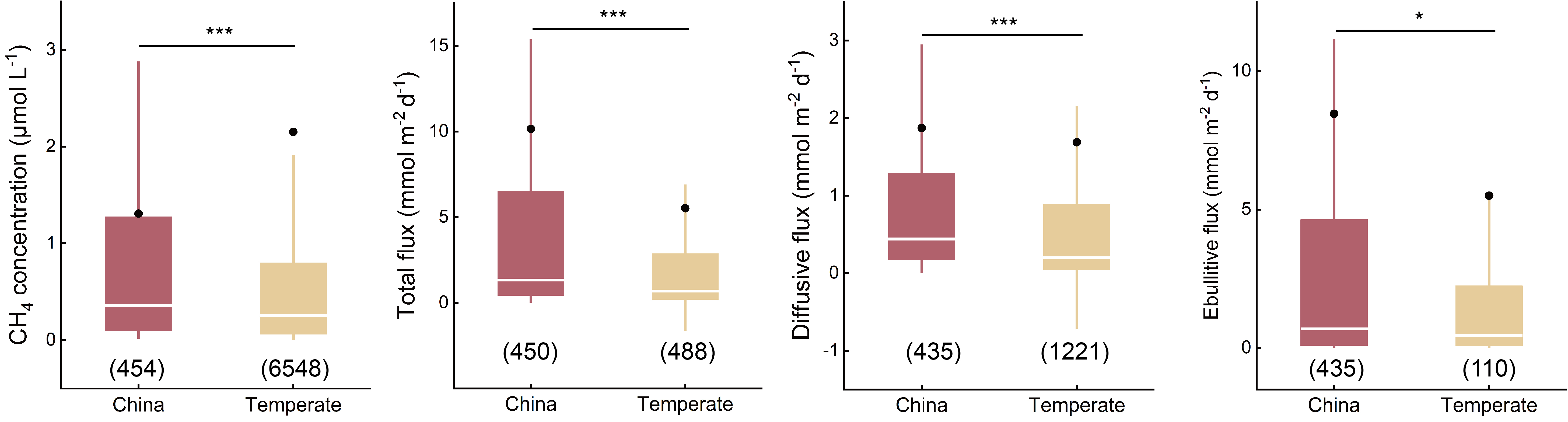
**Figure S3 Comparison of CH_4_ concentrations and fluxes between Chinese nutrient-enriched rivers and northern temperate pristine rivers.** Temperate pristine rivers are those outside of urban settlements, croplands, and densely populated regions (i.e. > 20 people km^-2^) in three northern temperate continents (i.e. Asia, Europe, and North America). White lines and black dots represent median and mean values of the group. Asterisks above the boxes indicate the significant difference between groups, which was tested with the two-sided Wilcoxon rank-sum test. Significance levels are represented as follows: ***, *p* < 0.001; **, *p* < 0.01; *, *p* < 0.05.


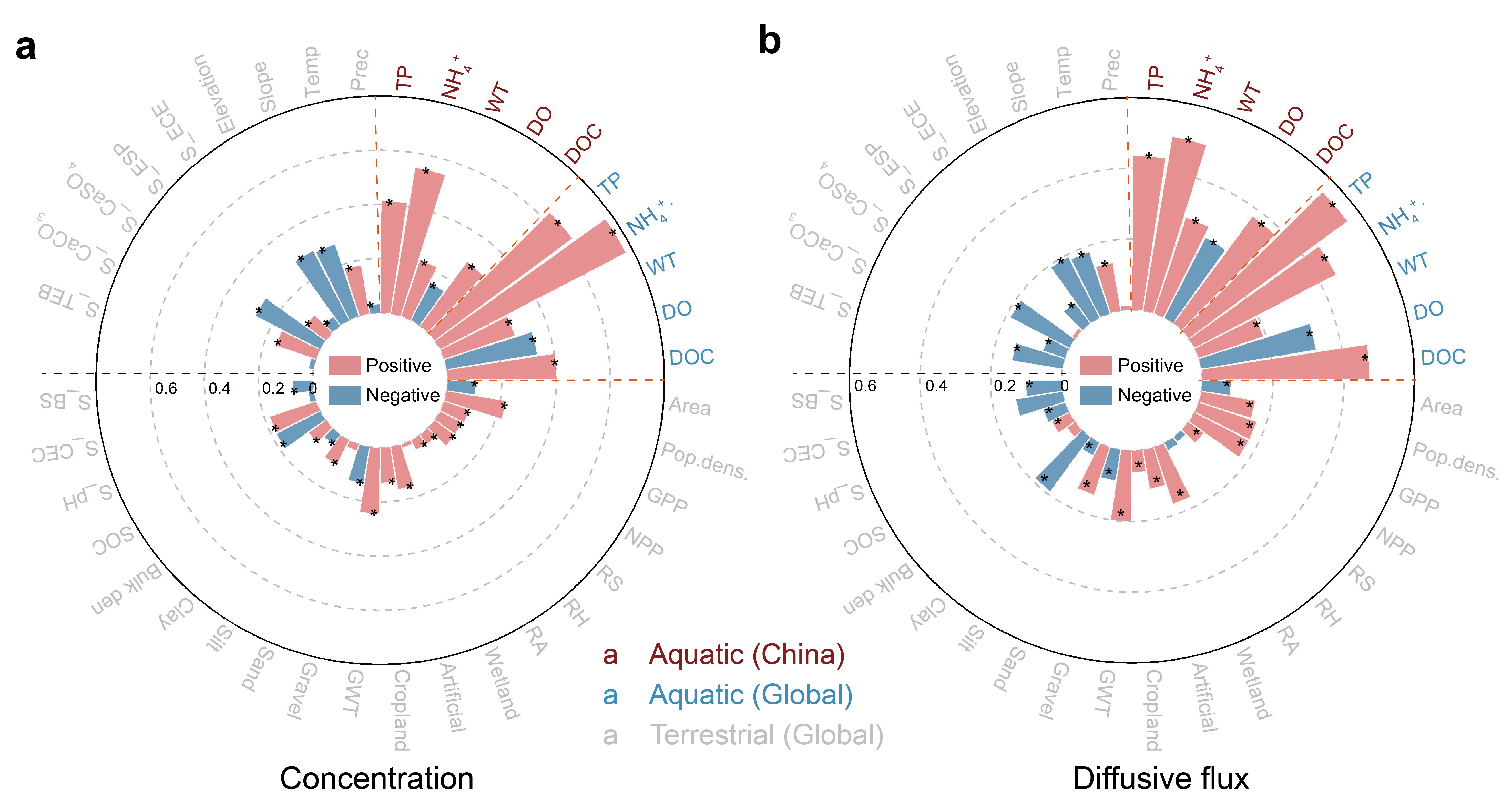
**Figure S4 Standardized regression coefficients between predictors and CH_4_ concentration and diffusive flux in Chinese dataset and human-impacted dataset of updated GRiMeDB.** All variables were standardized using the Z-score normalization method before standardized linear regressions. Bar height corresponds to absolute value of the standardized regression coefficients. Bars are colour-coded by their signs (positive or negative). Asterisks above the bars indicate the statistical significance of the regressions, with a *p*-value < 0.05. Regression analysis was performed on the aquatic variables across China dataset, aquatic variables across global human-impacted dataset, and terrestrial variables across global human-impacted dataset, respectively. TP, total phosphorus; NH_4+_, ammonium nitrogen; WT, water temperature; DO, dissolved oxygen; DOC, dissolved organic carbon; Area, catchment area; Pop. dens., population density; GPP, gross primary productivity; NPP, net primary productivity; RS, soil respiration; RH, heterotrophic respiration; RA, autotrophic respiration; Wetland, wetland proportion; Artificial, artificial land proportion; Cropland, cropland proportion; GWT, groundwater table; Gravel, soil gravel proportion; Sand, soil sand proportion; Silt, soil silt proportion; Clay, soil clay proportion; Bulk den, bulk density; SOC, soil organic carbon; S_pH, soil pH; S_CEC, soil cation exchange capacity; S_BS, soil base saturation; S_TEB, soil total exchangeable bases; S_CaCO_3_, soil calcium carbonate content; CaSO_4_, soil gypsum content; S_ESP, soil exchangeable sodium percentage; S_ECE, soil electric conductivity.


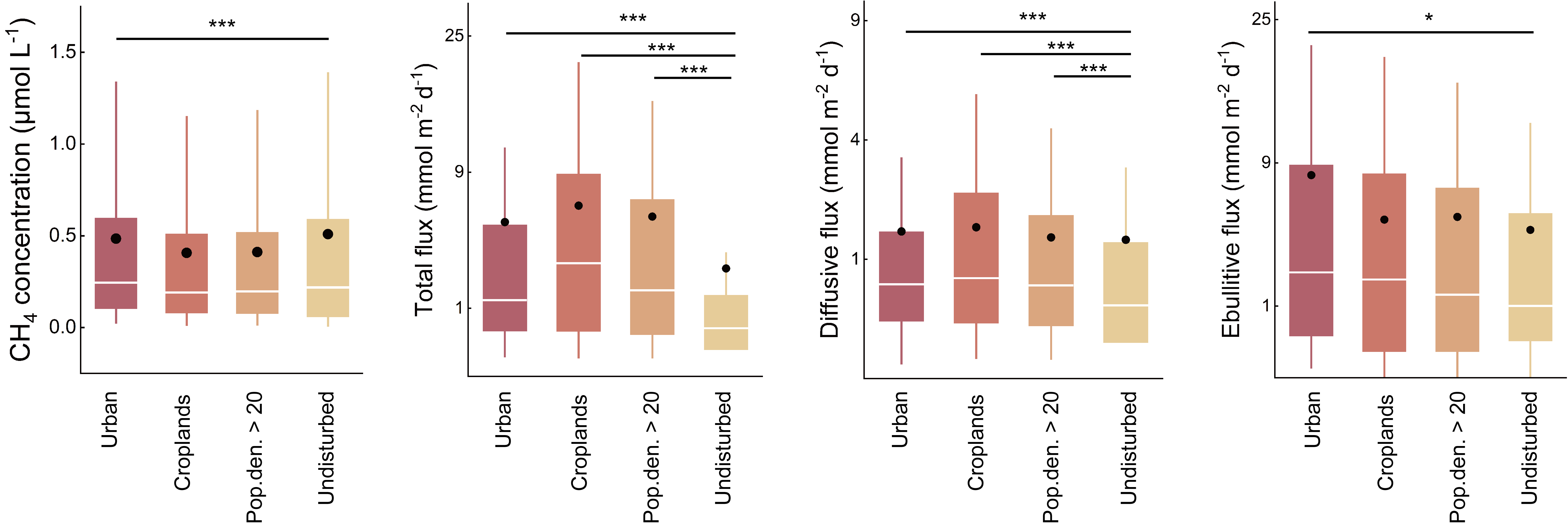
**Figure S5 Comparison of CH_4_ concentrations and fluxes across human disturbance types in updated GRiMeDB.** Human-impacted rivers are those that drain urban settlements, croplands, and densely populated regions (i.e. > 20 people km^-2^). Rivers outside of these regions are considered undisturbed. In each box, white solid line represents the median value and black dot denote the average. Asterisks above the bars indicate the significant difference among groups, which was tested with the two-sided Wilcoxon rank-sum test. Significance levels are represented as follows: ***, *p* < 0.001; **, *p* < 0.01; *, *p* < 0.05.

**
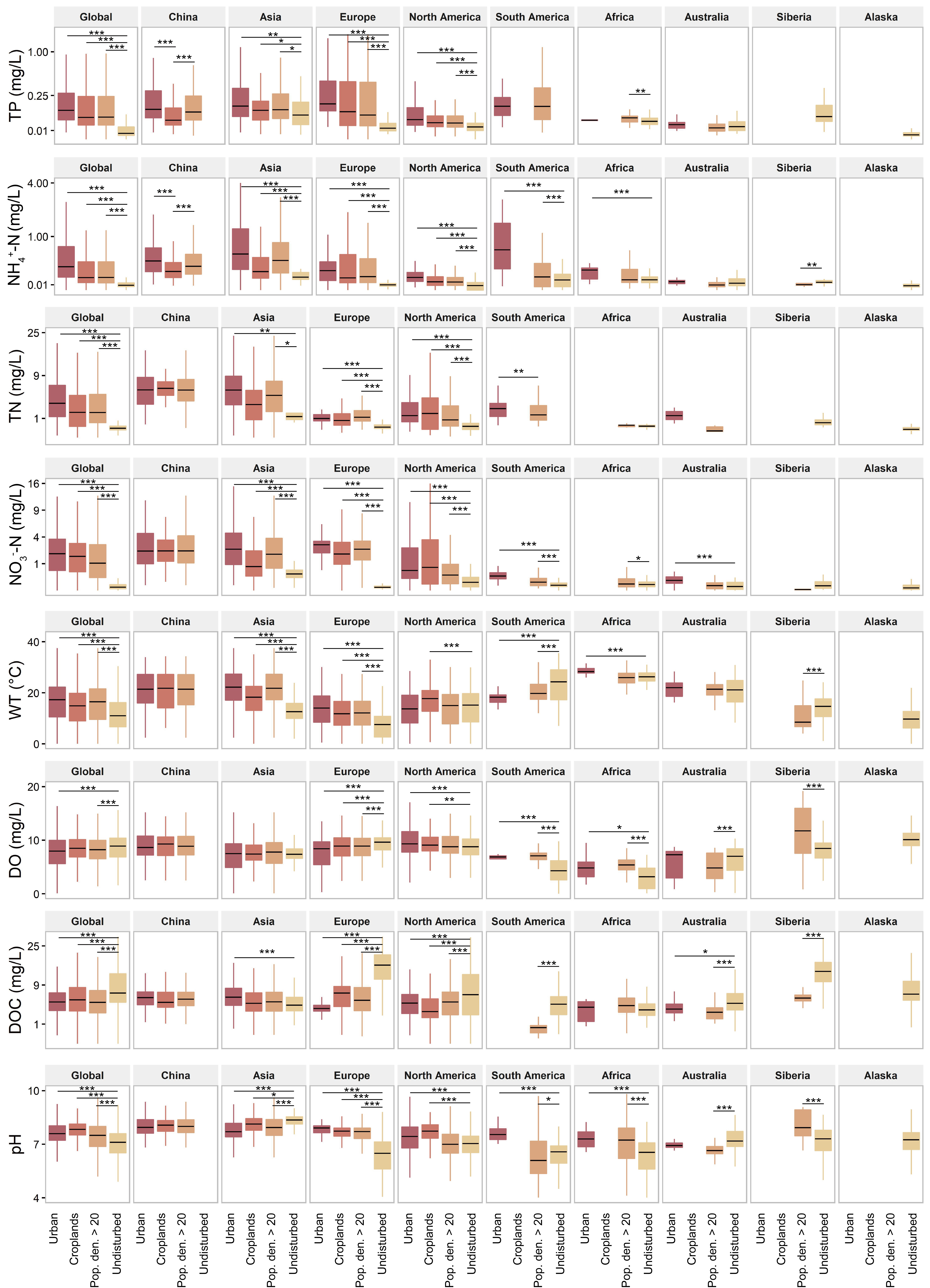
Figure S6 Comparison of eight aquatic variables across human disturbance types in updated GRiMeDB.** Human-impacted rivers are those that drain urban settlements, croplands, and densely populated regions (i.e. > 20 people km^-2^). Rivers outside of these regions are considered undisturbed. Black solid line in each box represents the median value. Asterisks above the bars indicate the significant difference among groups, which was tested with the Wilcoxon rank-sum test. Significance levels are represented as follows: ***, *p* < 0.001; **, *p* < 0.01; *, *p* < 0.05.


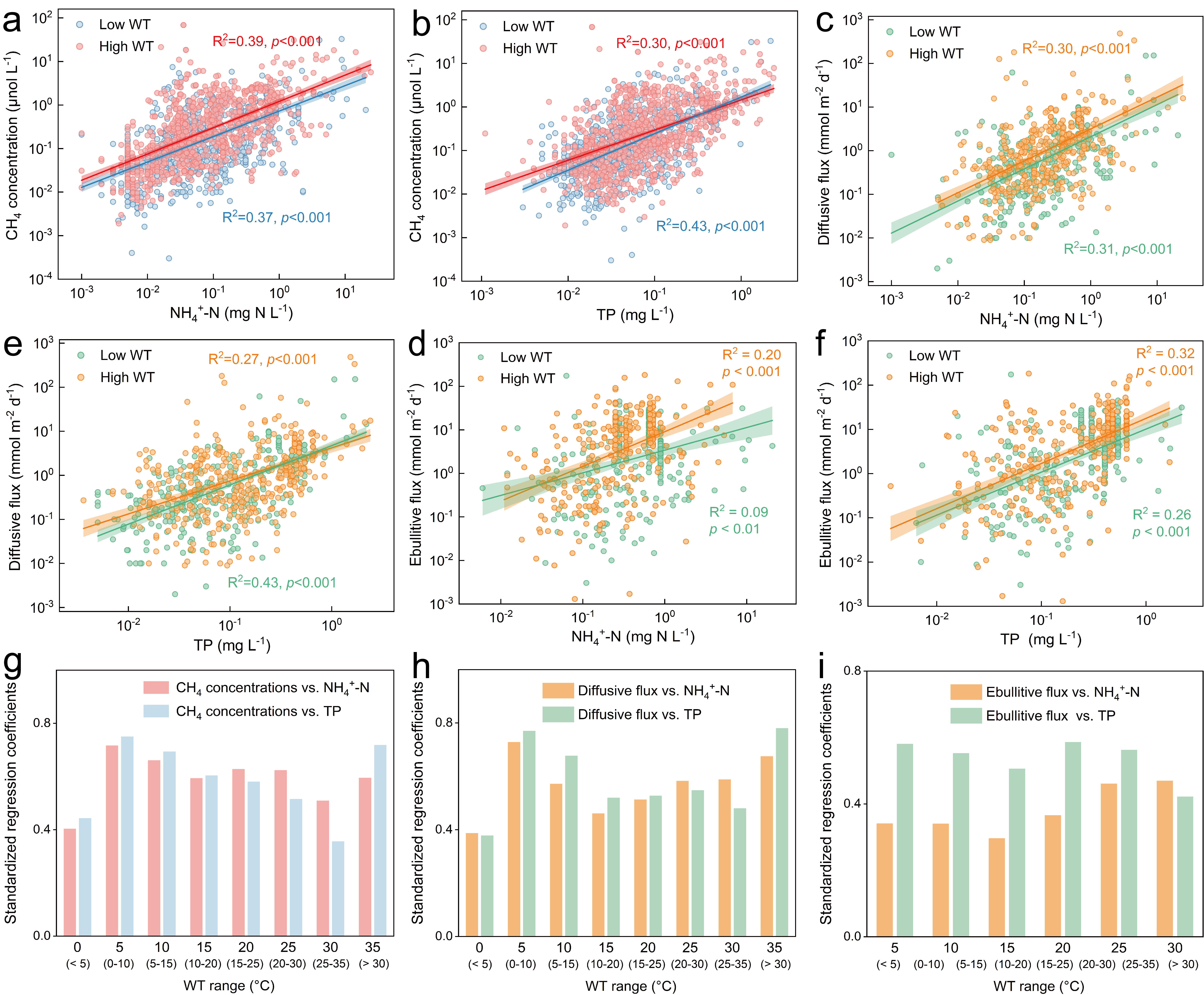
**Figure S7 Synergistic effects of water temperature and nutrient concentrations on CH_4_ concentration and flux.** Data from human-impacted rivers were divided into high- and low-temperature subsets based on the median (~18 °C). Linear regressions were performed between nutrient concentrations and CH_4_ concentration (**a, b**), diffusive flux (**c, d**), and ebullitive flux (**e, f**) across temperature groups, respectively. Linear regressions for each subgroup are shown in distinct colors, with shaded bands representing the 95% confidence interval. (**g-i**) Standardized regression coefficients derived from moving-window analyses (10 °C intervals).


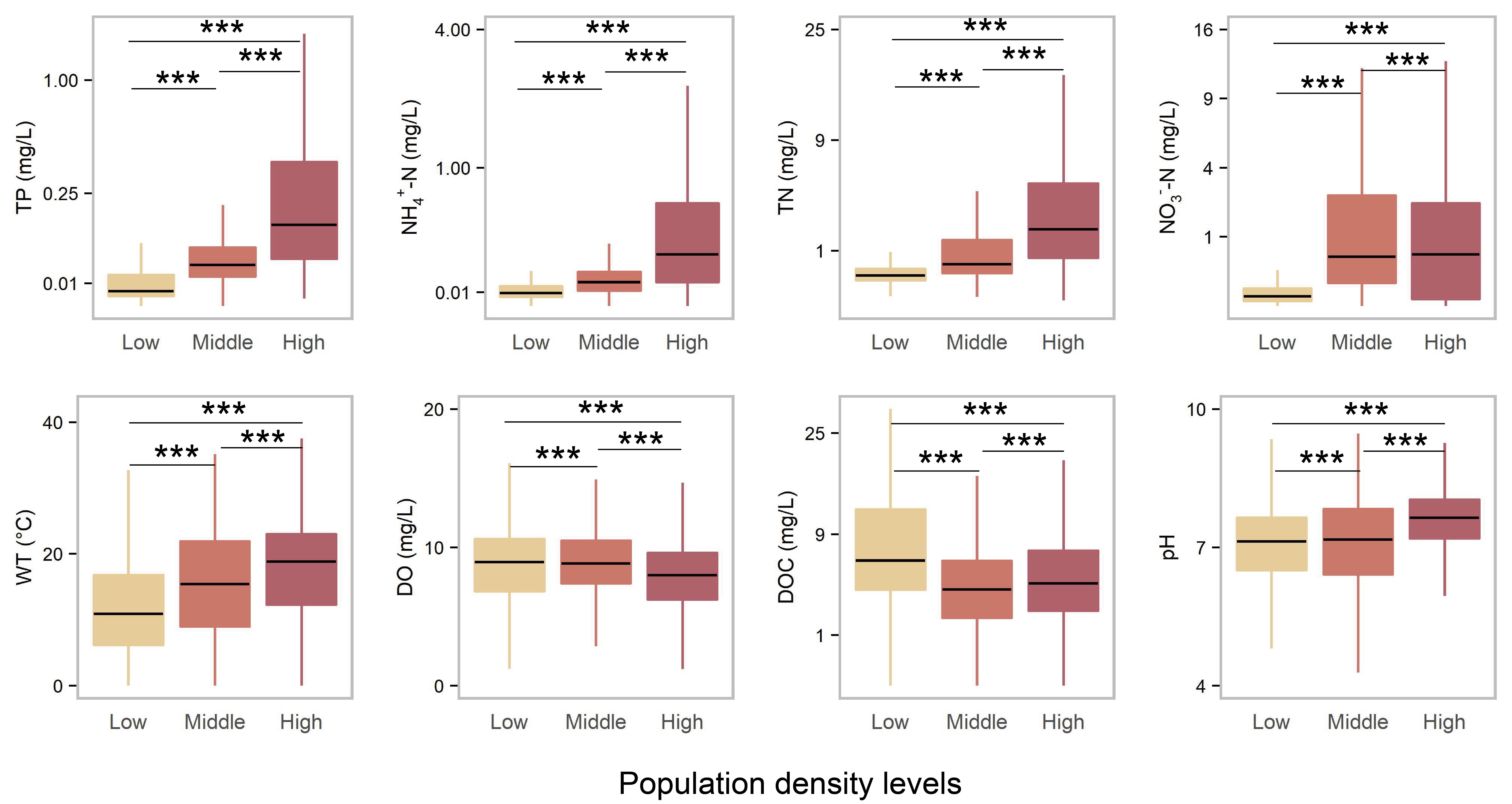
**Figure S8 Comparison of eight aquatic variables across different population density levels in updated GRiMeDB.** Data were classified into three population density levels based on the population density (low, population density < 20 people km^-2^; middle, 20 ≤ population density < 100 people km^-2^; high, population density ≥ 100 people km^-2^). Black solid line in each box represents the median value. Asterisks above the bars indicate the significant difference among groups, which was tested with the Wilcoxon rank-sum test. Significance levels are represented as follows: ***, *p* < 0.001; **, *p* < 0.01; *, *p* < 0.05.


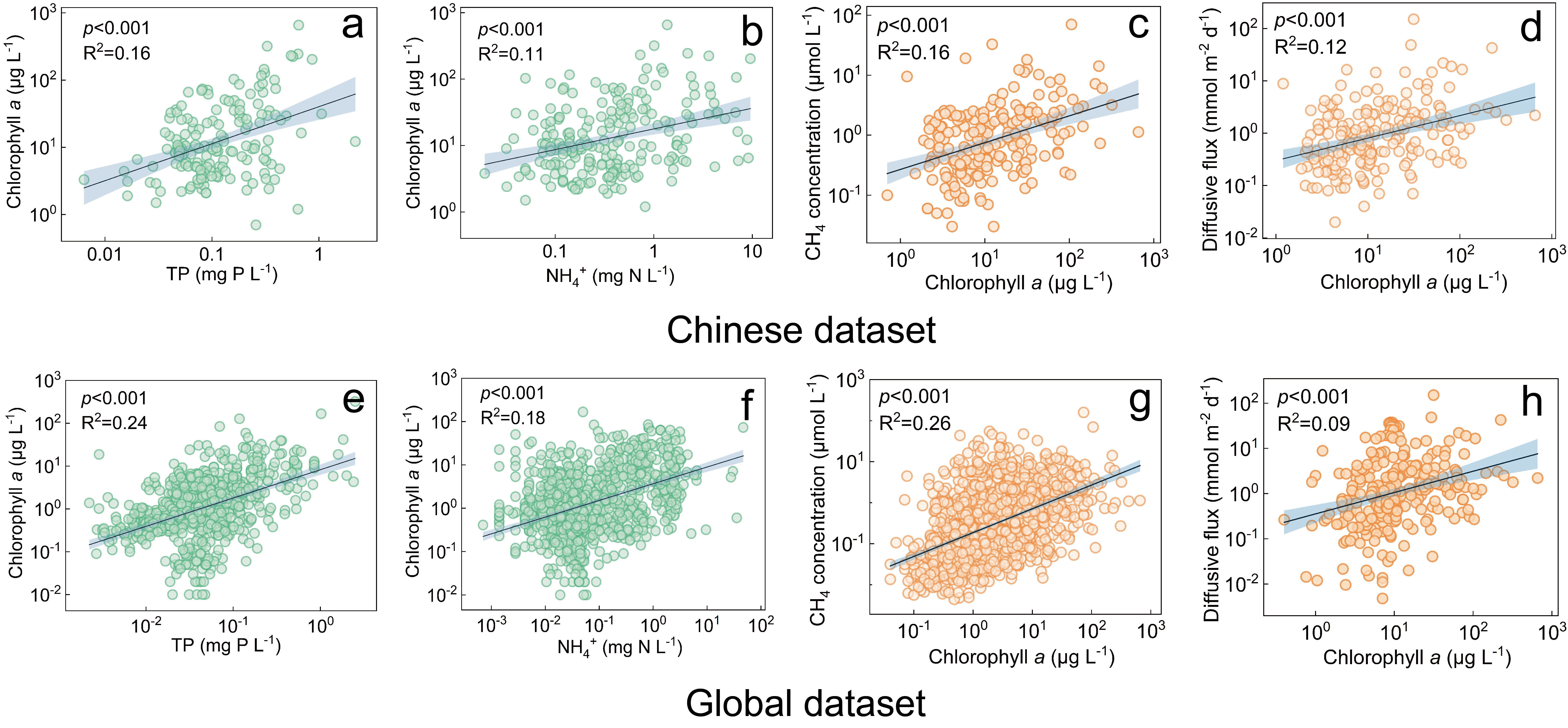
**Figure S9 Relationships between CH_4_ concentration and flux, chlorophyll *a* concentration, and nutrient concentration (TP and NH_4_^+^-N) in Chinese rivers and global human-impacted rivers.** The black line represents the fit of a linear regression, and R^2^ is the coefficient of determination.

**
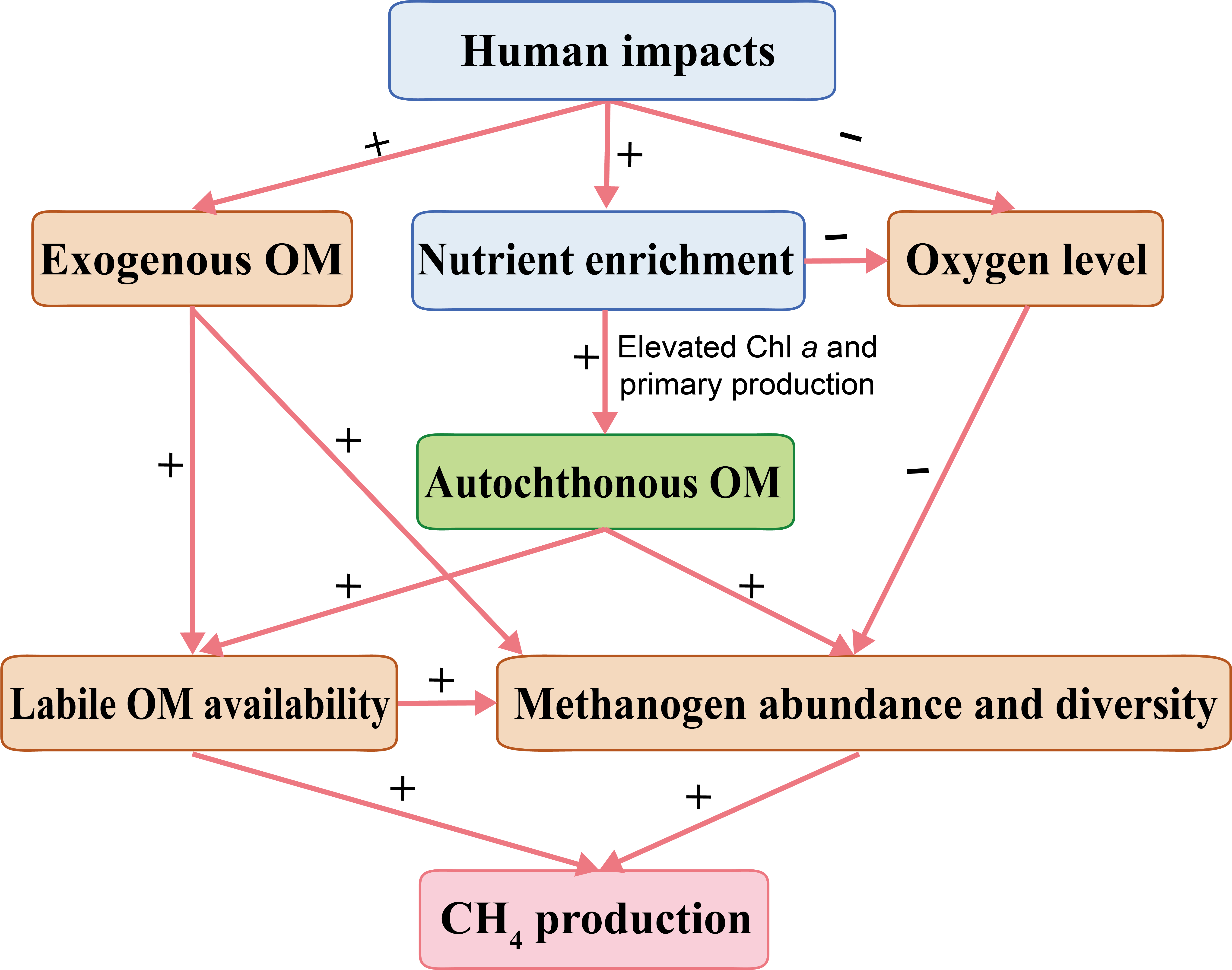
Figure S10 Schematic of the pathways through which nutrient enrichment amplifies CH_4_ production in human-impacted rivers.** Pathways marked “+” and “-” denote positive and negative effects, respectively.


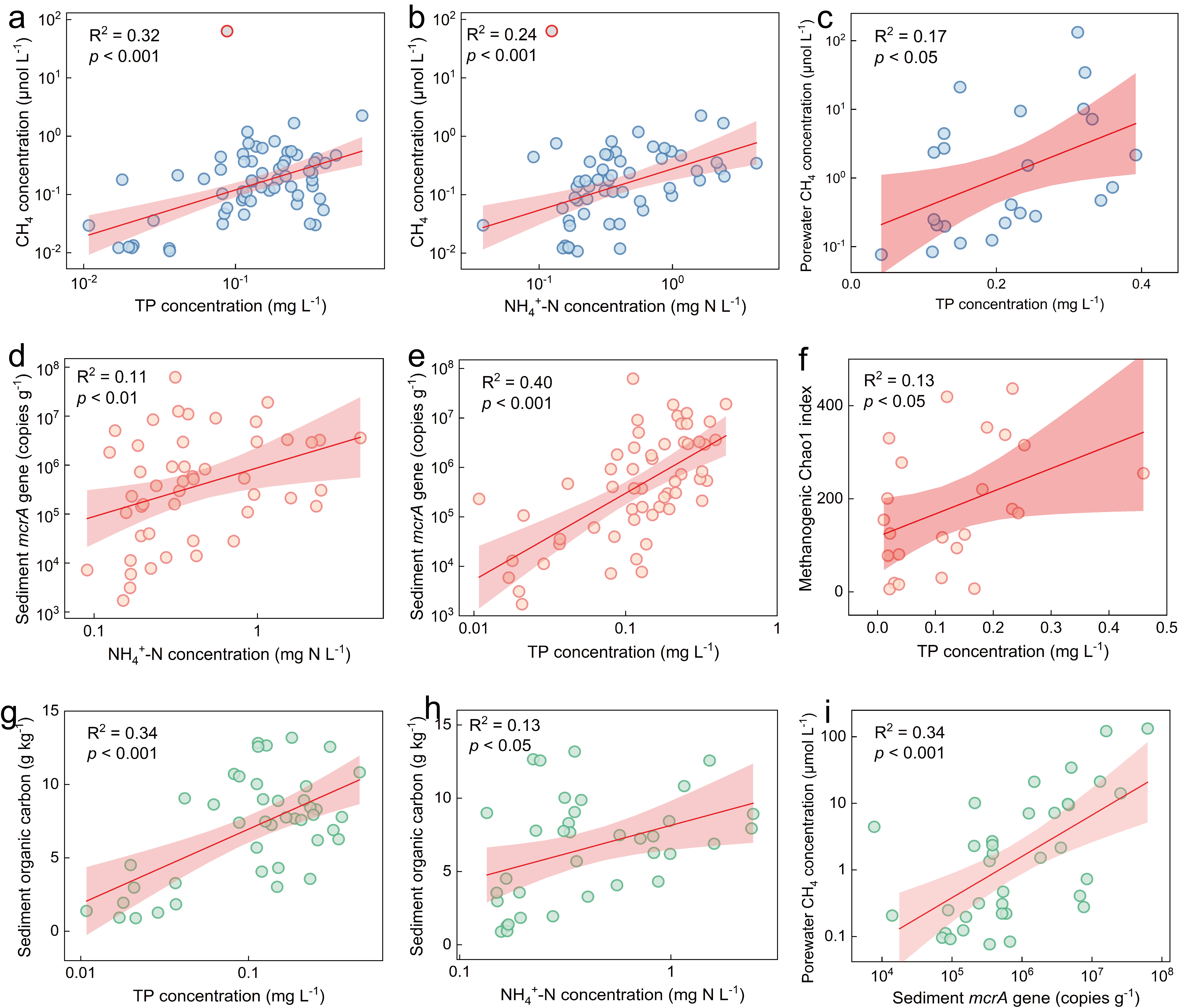
**Figure S11 Relationships among CH_4_ concentration, nutrient concentrations, sediment organic carbon, sediment *mcrA* gene abundance and methanogenic Chao1 index from the 2021 field campaign of the Yellow River Basin.** Sediment *mcrA* gene abundance and methanogenic chao represent the abundance and diversity of methanogenic microorganisms in sediment, respectively. The red solid lines represent the fitted linear regressions, with shaded bands representing the 95% confidence interval. The R^2^ and *p*-value of each fit are shown in each panel.



**Figure S12 Standardized regression coefficients between six aquatic variables and CH_4_ concentration and fluxes in rivers across the global and continental (sub-)datasets.** All variables were standardized using the Z-score normalization method before standardized linear regressions. (Sub-)Datasets across different continents: gl, global; ar, Alaska; si, Siberia; as, Asia; eu, Europe; na, North America; af, Africa; sa, South America; au, Australia. Figures below the bars in panels represent the sample size used in the regression analysis, and the asterisks above the bars indicate the statistical significance of the regression results. Significance levels are represented as follows: ***, *p* < 0.001; **, *p* < 0.01; *, *p* < 0.05.


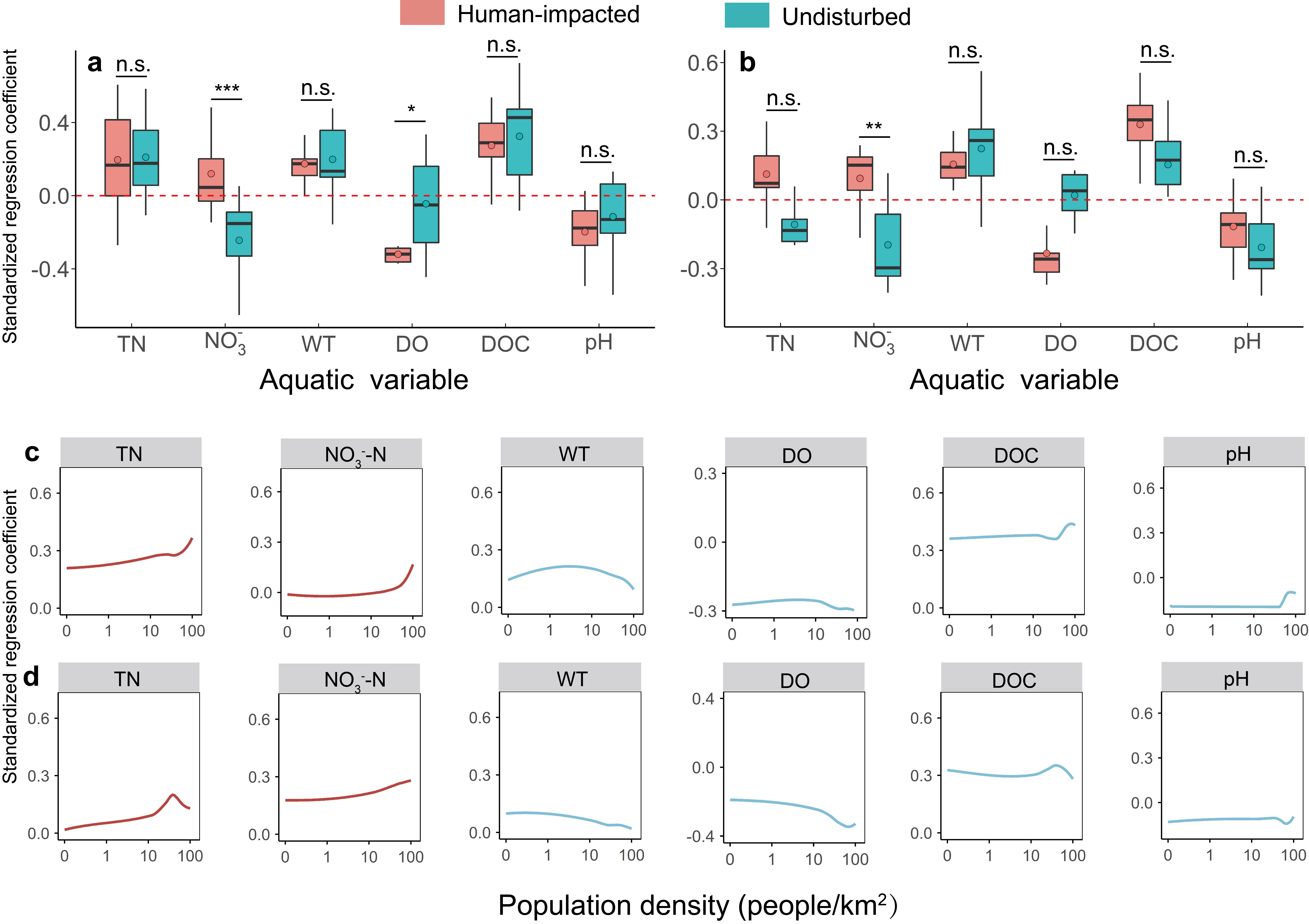
**Figure S13 Correlations between other six aquatic variables and CH_4_ concentration and fluxes. a**, **b** Standardized regression coefficients between six aquatic variables and CH_4_ concentration (**a**) and fluxes (**b**) across human-impacted and undisturbed sub-datasets. All variables were standardized using the Z-score normalization method before standardized linear regressions. Data in each box include standardized regression coefficients from human-impacted and undisturbed sub-datasets. Box spans the 25th and 75th percentiles. In each box, solid line denotes the median value and the whiskers represent 1.5× the interquartile range. Statistical significance between groups was tested with the two-sided Wilcoxon rank-sum test. Significance levels are represented as follows: ***, *p* < 0.001; **, *p* < 0.01; *, *p* < 0.05. **c**, **d** Standardized regression coefficients between six aquatic variables and CH_4_ concentration (**c**) and fluxes (**d**) at different population density levels. The curves of the standardized regression coefficients have been smoothed using loess method.


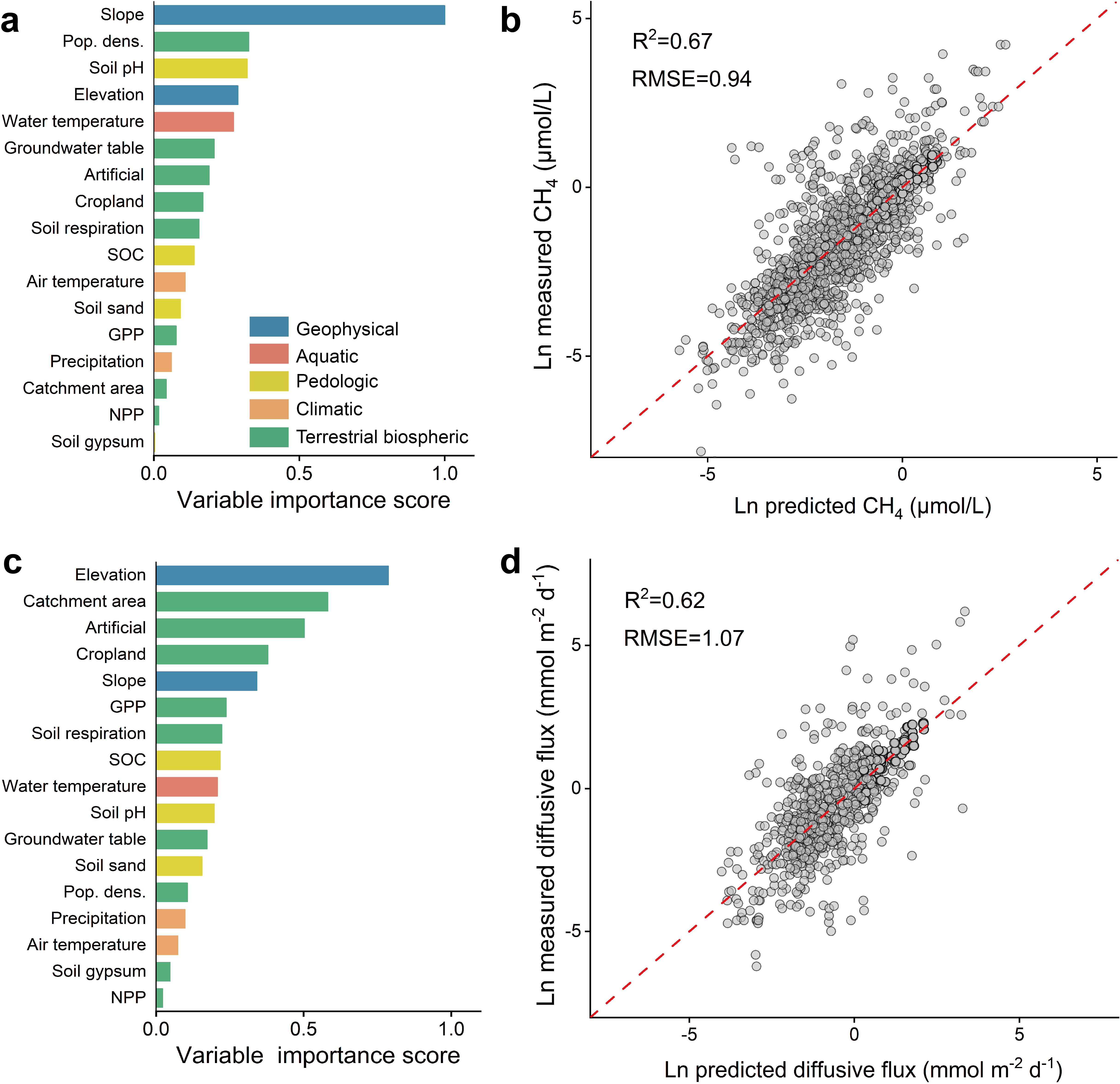
**Figure S14 Variable importance and model performance of global human-impacted rivers excluding aquatic nutrient variables.** Variable importance ranking and model performance of CH_4_ concentration (**a**, **b**) and diffusive flux (**c**, **d**) modelled by Random Forest algorithms. Dashed line represents the 1:1 line. R^2^ is the regression coefficient of the linear regression and RMSE is the root mean square error. Variables include slope, elevation, water temperature, soil gravel proportion (Soil gravel), soil pH, soil gypsum content (Soil gypsum), soil organic carbon (SOC), population density (Pop dens), catchment area, artificial land proportion (Artificial), cropland proportion (Cropland), gross primary production (GPP), net primary production (NPP), soil respiration, groundwater table, air temperature, and precipitation.


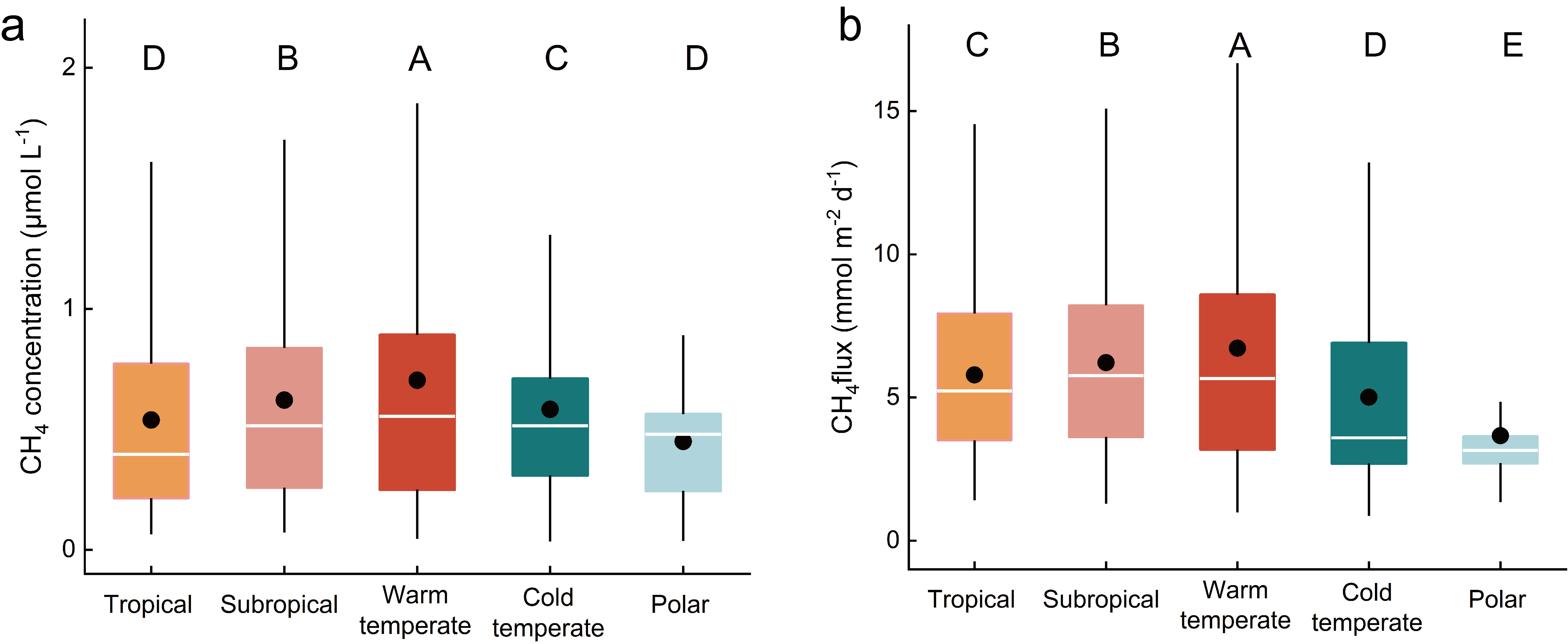
**Figure S15 Climatic patterns of predicted CH_4_ concentrations and fluxes from global human-impacted rivers.** In each box plot, box spans the 25th and 75th percentiles. Solid line denotes the median and black dot denotes the mean, with whiskers representing 1.5× the interquartile range. Box plots show CH_4_ concentrations and fluxes across five climatic zones, tropical (0–10°), subtropical (10–20°), warm temperate (20–40°), cold temperate (40–60°), and polar (>60°). Statistical significance between groups was tested with one-way ANOVA followed by Tukey's post hoc test. The different capitals above each subfigure represent significant difference at *p* < 0.05.


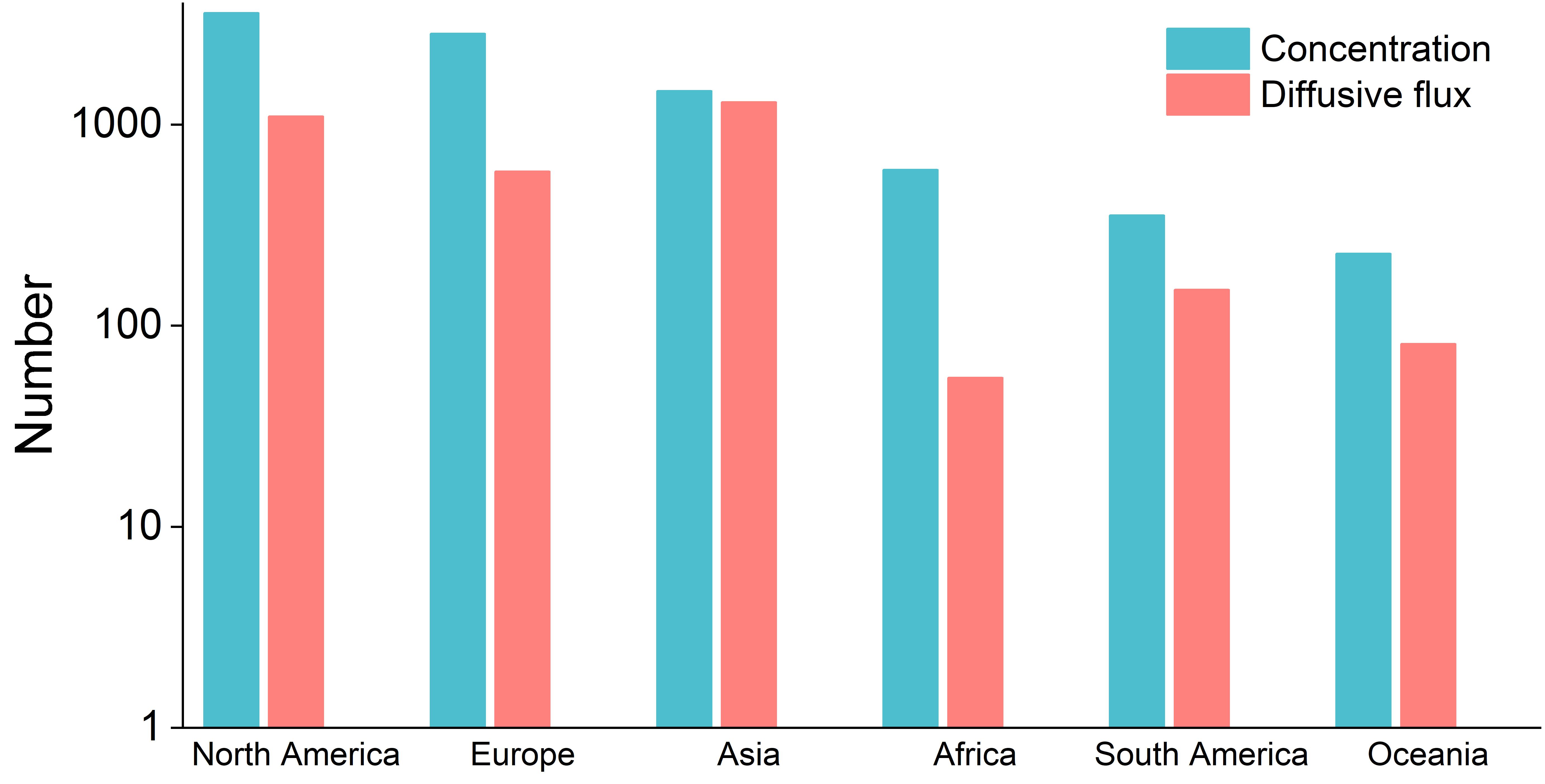
**Figure S16 Continental distributions of CH_4_ concentration and diffusive flux observations from global human-impacted rivers.**


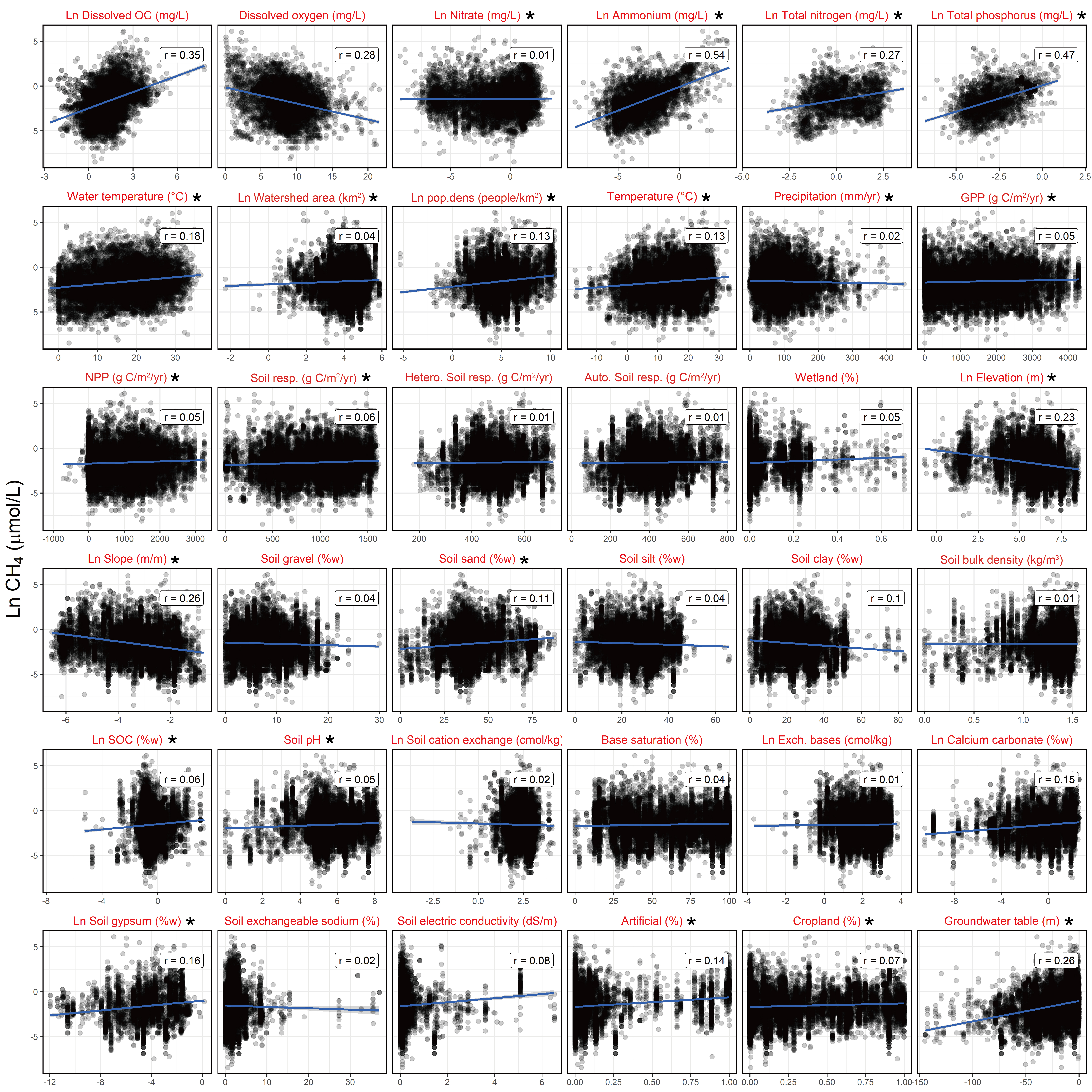
**Figure S17 Linear regressions between CH_4_ concentration and environmental predictors.** Reach-scale predictors include geophysical, climatic, edaphic, terrestrial biospheric variables, and aquatic attributes. Solid line represents the linear regression. Correlation coefficients of determination are shown in the plots. Variables marked with an asterisk were selected for subsequent machine learning modeling.


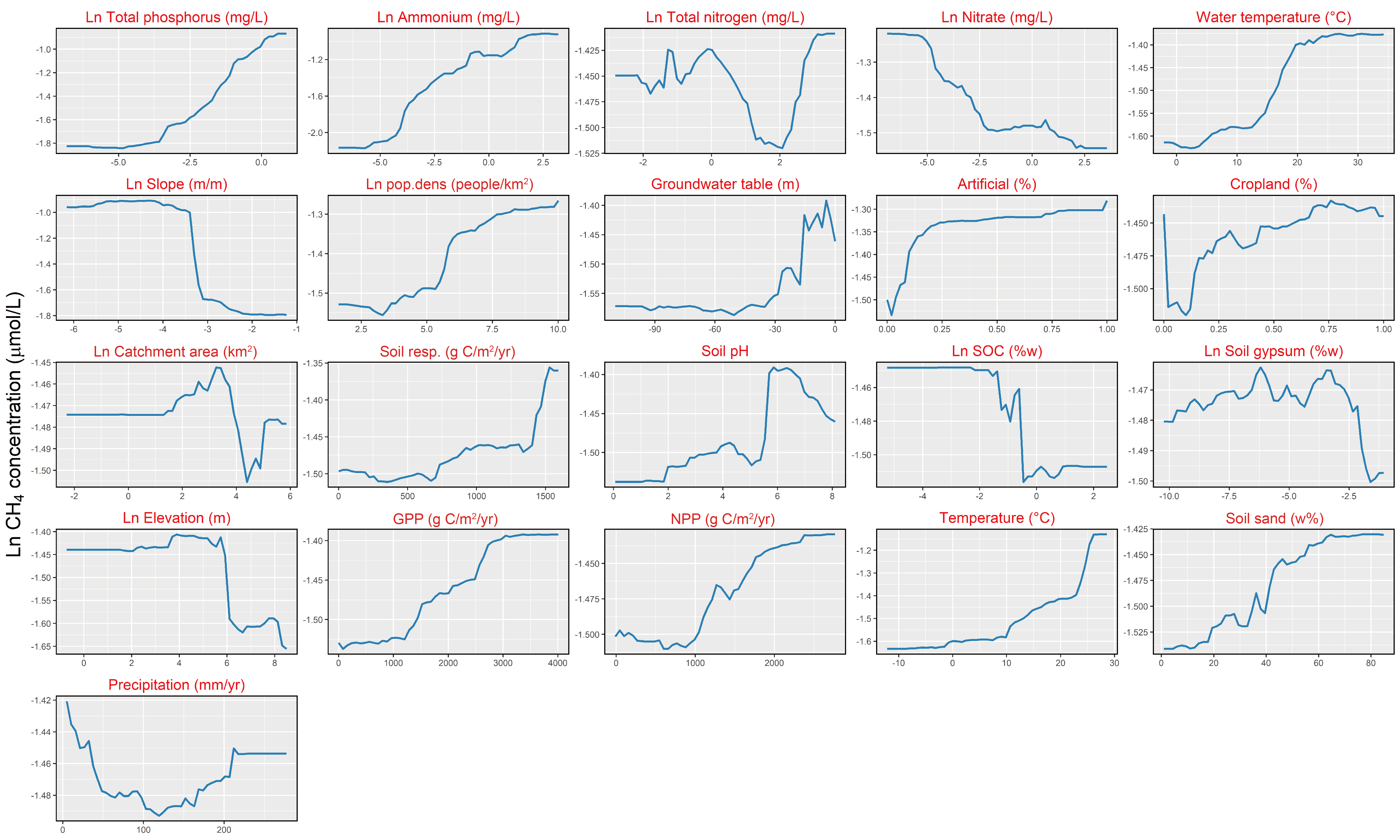
**Figu****re S18** **Partial dependence plot of CH_4_ concentration for the 21 reach-scale environmental predictors incorporated into the machine learning models.**


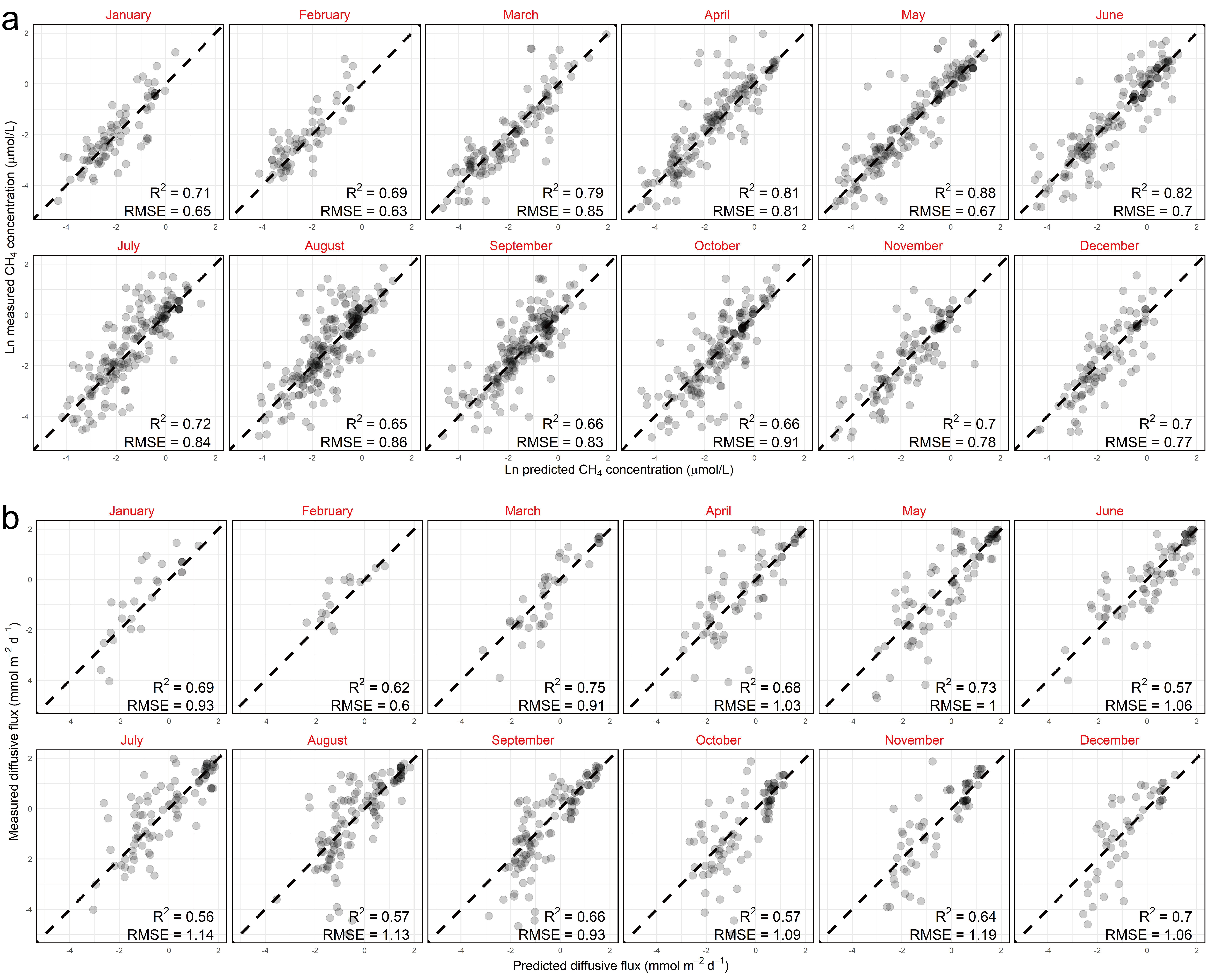
**Figure S19 Monthly model performance for CH_4_ concentration (a) and diffusive flux (b).** The results are the averages of the three machine learning outputs. Dashed line represents the 1:1 line. R^2^ is the coefficient of determination of the linear regression and RMSE is the root mean square error.


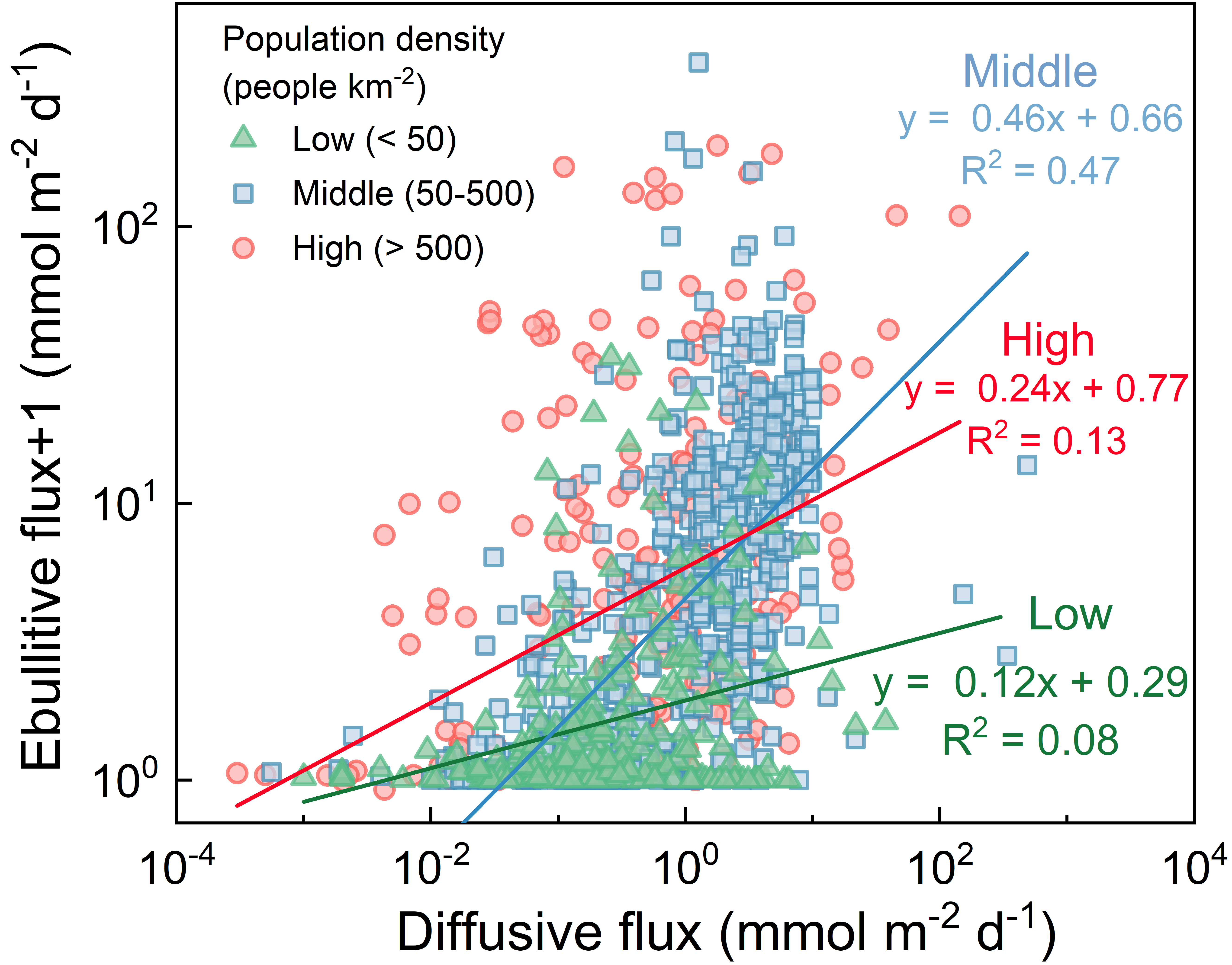
**Figure S20 Relationship between ebullitive and diffusive CH_4_ fluxes across river segments grouped by population density.** Data are stratified into three groups based on population density: low (< 50 people km^-2^), middle (50–500 people km^-2^), and high (> 500 people km^-2^) population density categories, with a linear fit applied to each group.

**Figure S21**
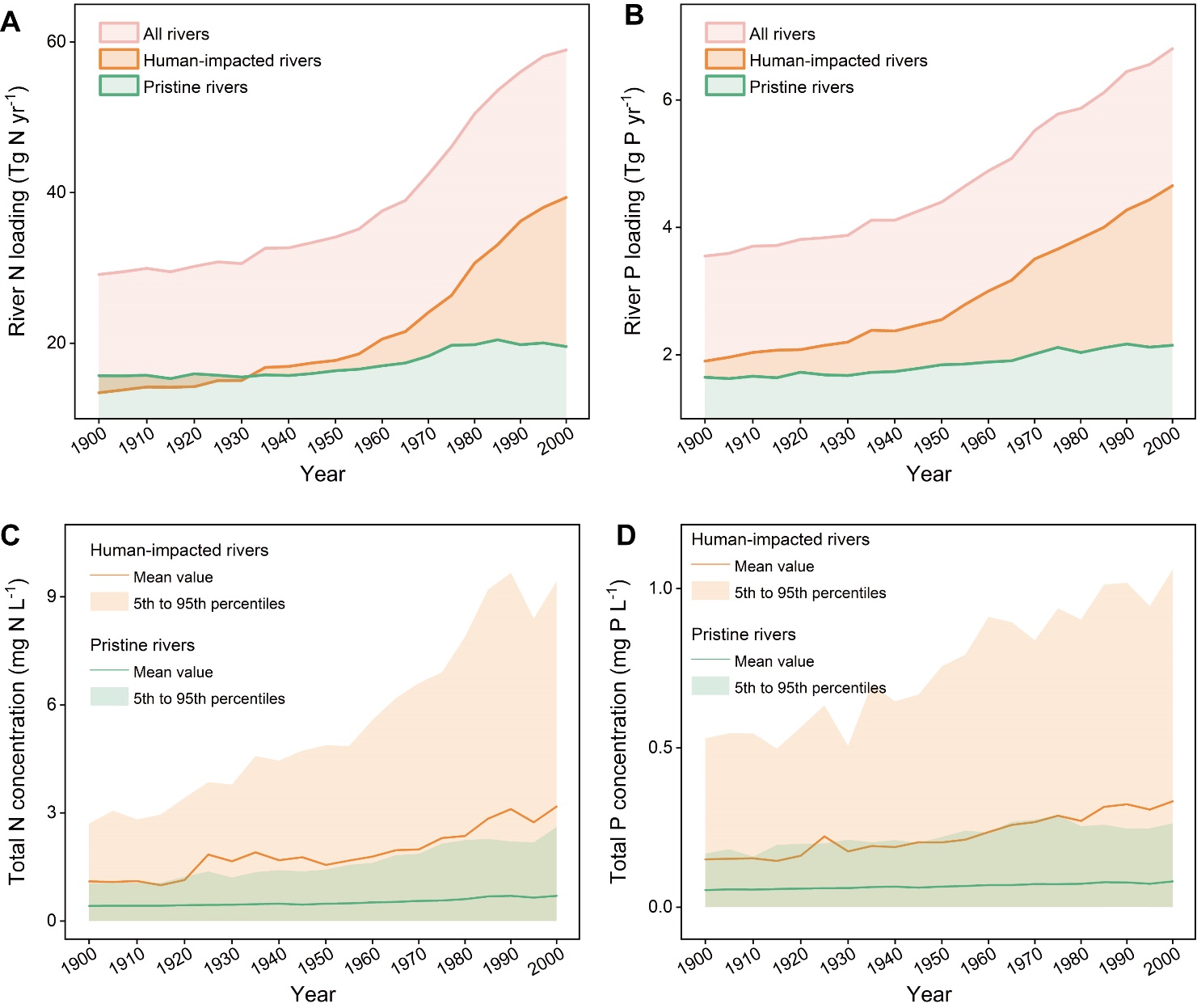
**Nitrogen and phosphorus loading as well as their concentrations in human-impacted rivers and undisturbed rivers from 1900 to 2000.** Human-impacted rivers are those that drain urban settlements, croplands, and densely populated regions (i.e. > 20 people km^-2^). Rivers outside of these regions are considered undisturbed. Nitrogen and phosphorus data is sourced from the Integrated Model to Assess the Global Environment–Global Nutrient Model (IMAGE-GNM)[[19](#_ENREF_19),[20](#_ENREF_20)].


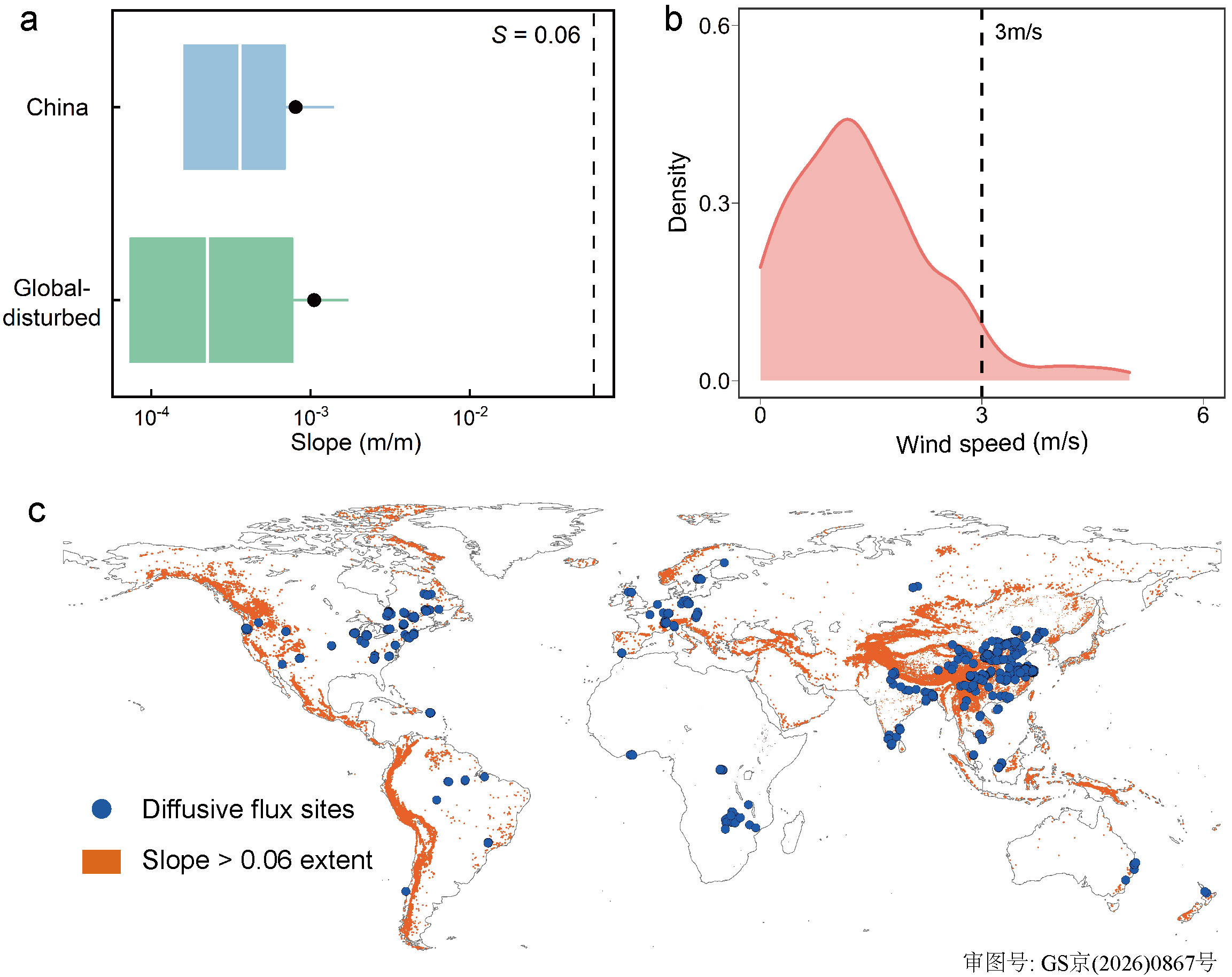
**Figure S22 Slope and wind speed at sampling sites from Chinese and global disturbed rivers.** **a**, Comparative box plot of channel slopes. Box spans the 25th and 75th percentiles. Solid line denotes the median and the whiskers represent 1.5× the interquartile range. The vertical dashed line marks the mean slope of high-energy streams (0.06 m/m). **b**, Distribution density of wind speed at Chinese river sites. The vertical dashed line indicates 3 m/s. **c**, Global map showing regions with slopes > 0.06 m/m and the geographical distribution of diffusive flux sampling sites.


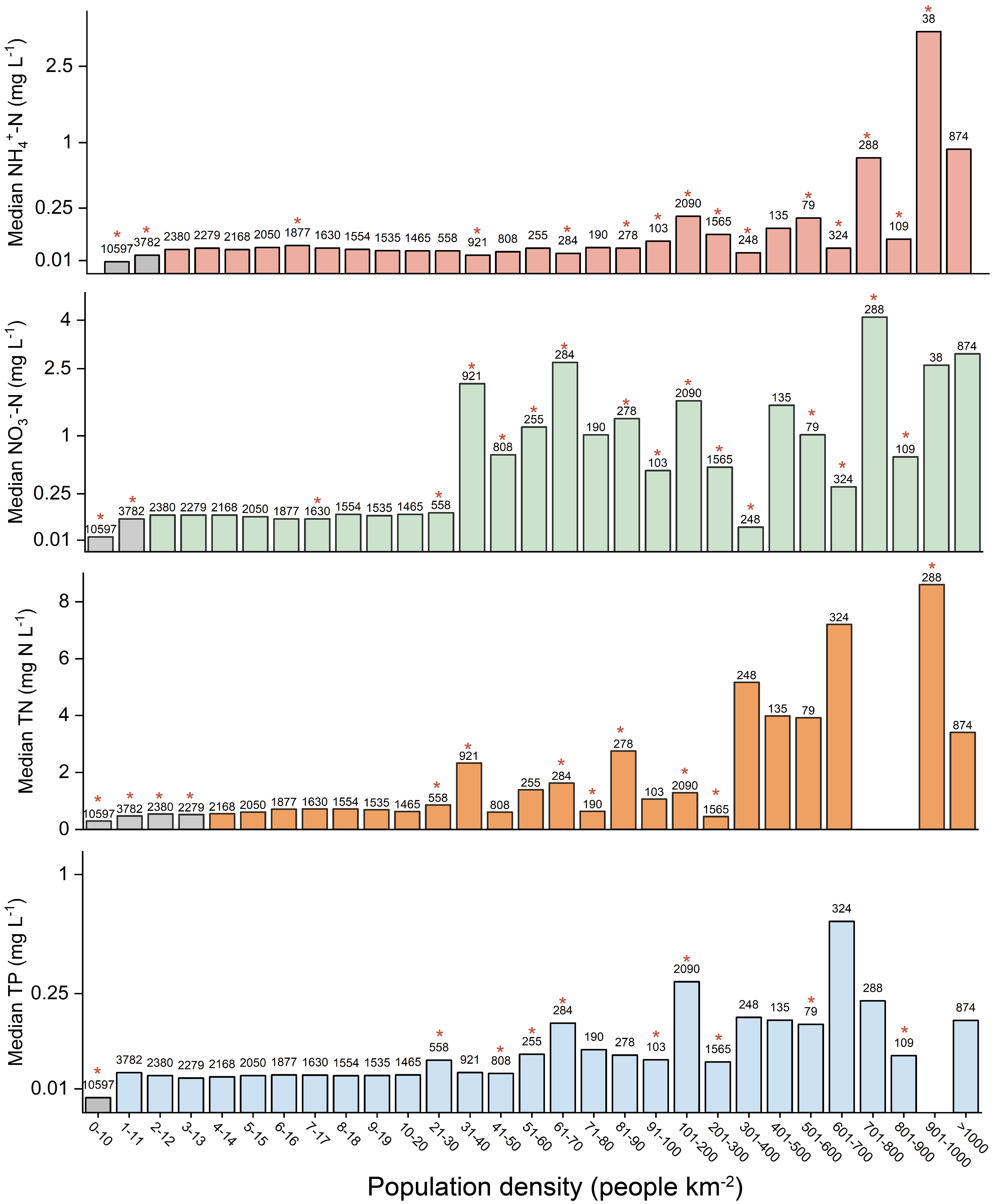
**Figure S23 Moving window analysis of four dissolved nutrient concentration (TP, NH_4_^+^-N, TN, NO_3_^-^-N) across different population density subsets.** Each window included a subset of dissolved nutrient concentrations within a 10 and 100 interval when population density < 100 and > 100 people km^-2^, respectively, and moved forward by 1 (population density at 0-10), 10 (population density at 10-100), 100 (population density at 100-1000) steps. The asterisks above bars represent the significant difference in nutrient concentrations between the subset and the following subset at *p* < 0.05 through Wilcoxon rank-sum test. Figure above each bar represents the data size within each population density subset. The grey bars are identified as undisturbed subsets of population density through our discerning criterion (See ***Supporting Methods 6***).



**Figure S24 Relationships between CH_4_ and nutrient concentrations in human-impacted rivers defined by different population density thresholds.** Linear regressions were performed for data stratified by increasing population density thresholds (>0, >10, >20, >50, and >100 people km^-2^). Sample size (n) and coefficient of determination (R^2^) are annotated for each subgroup. Data points are color-coded by population density.


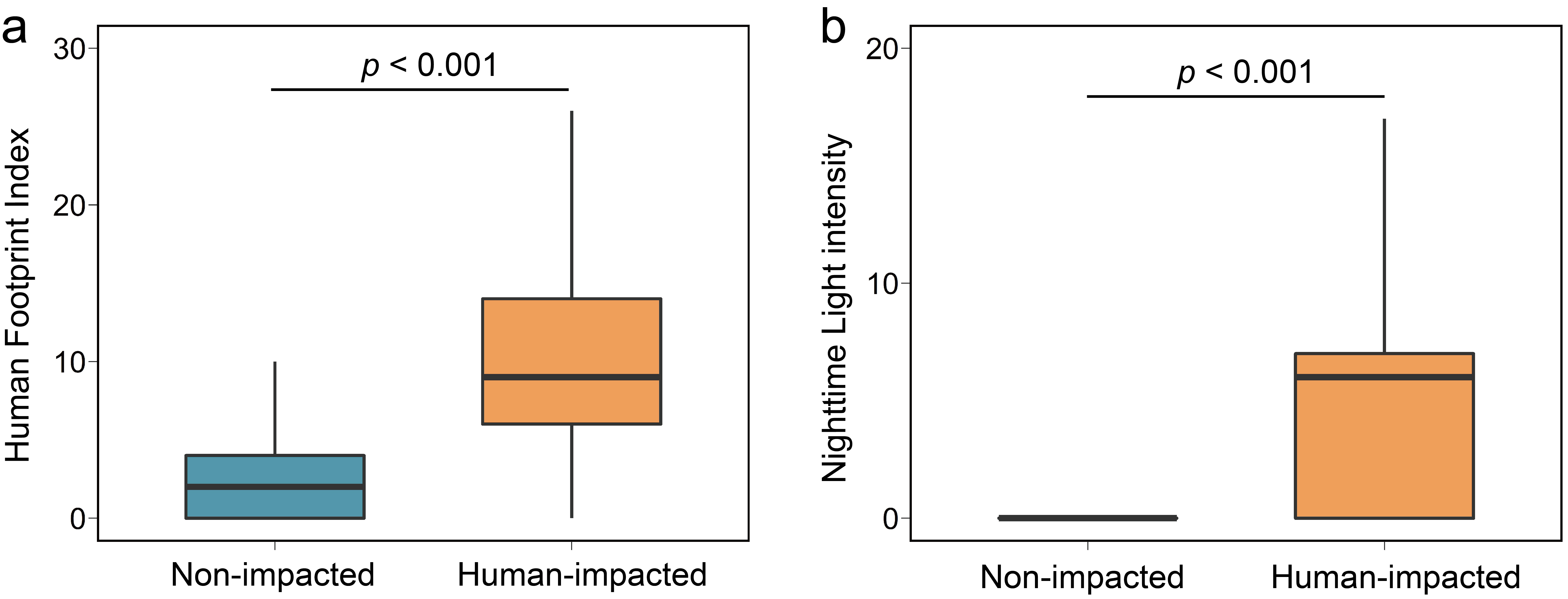
**Figure S25 Comparison of Human Footprint Index and Nighttime Light intensity between** **defined** **non-impacted and human-impacted regions globally.** Box spans the 25th and 75th percentiles. Solid line denotes the median and the whiskers represent 1.5× the interquartile range. Statistical significance between groups was tested with the two-sided Wilcoxon rank-sum test.


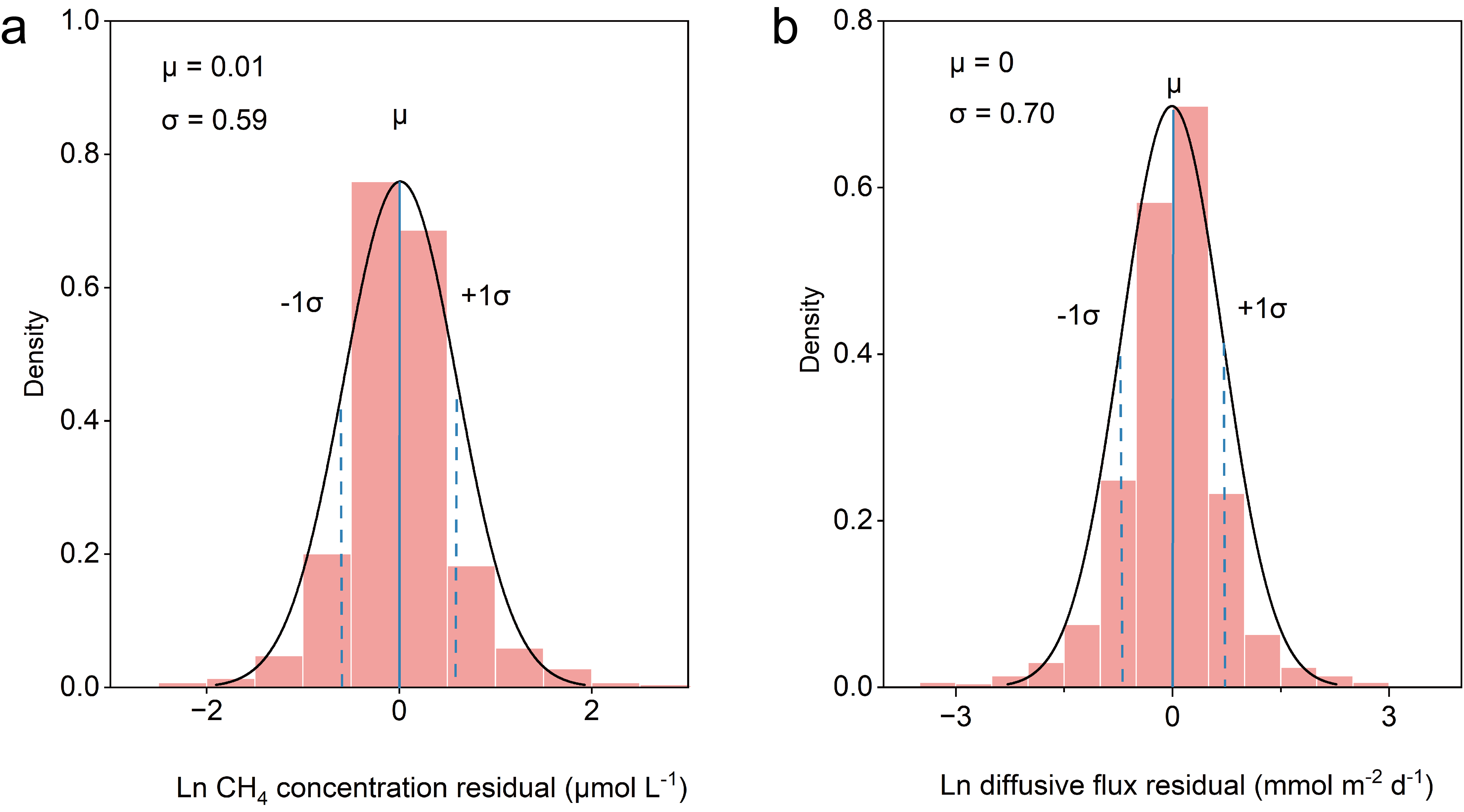
**Figure S26 The fitting of the model residual to a log-normal distribution in terms of ln CH_4_ concentration and diffusive flux.** The residuals represent the differences between the measured values and the predicted values, which are averaged from three machine learning algorithms. The model residuals are fitted to a normal distribution and calculate the error at one standard deviation (1σ).

# Supporting Tables

**Table S1 Model performance of three machine learning algorithms when excluding and including aquatic nutrient variables**

| Algorithms | Variables | Performance index | Random forest | XGBoost | SVM |
| --- | --- | --- | --- | --- | --- |
| CH_4_ concentration | Excluding  nutrients | R^2^ | 0.67 | 0.67 | 0.58 |
|  |  | RMSE | 0.94 | 0.93 | 1.06 |
|  | Including nutrients | R^2^ | 0.77 | 0.77 | 0.70 |
|  |  | RMSE | 0.78 | 0.79 | 0.90 |
| Diffusive flux | Excluding  nutrients | R^2^ | 0.62 | 0.62 | 0.57 |
|  |  | RMSE | 1.07 | 1.06 | 1.14 |
|  | Including nutrients | R^2^ | 0.71 | 0.71 | 0.67 |
|  |  | RMSE | 0.94 | 0.93 | 1.01 |

**Table S2 Parameter tuning and optimal values of random forest, XGBoost, and SVM algorithms for concentration and diffusive flux models**

| Algorithms | Parameters  (Ranges) | Optimal value | |
| --- | --- | --- | --- |
|  |  | Concentration model | Diffusive flux model |
| Random forest | mtry  (1-20) | 7 | 7 |
|  | ntree  (100-1000) | 400 | 500 |
|  | nodesize (1-10) | 1 | 5 |
| XGBoost | nrounds  (100-500) | 200 | 100 |
|  | max_depth  (3, 6, 9) | 9 | 6 |
|  | eta  (0.01, 0.1, 0.2) | 0.1 | 0.1 |
|  | gamma  (0, 0.1, 0.2) | 0.1 | 0.2 |
|  | colsample_bytree  (0.1-1) | 0.5 | 0.5 |
|  | min_child_weight  (1, 5, 10) | 10 | 10 |
| SVM | sigma  (0.1,1,10,100) | 10 | 0.1 |
|  | C  (0.1,1,10,100) | 0.1 | 1 |

**Table S3 Sensitivity test for model performance by replacing the four most predictive variables with aquatic nutrient variables in random forest models**

| Model | Replaced variables | Model performance | | | |
| --- | --- | --- | --- | --- | --- |
|  |  | Before replacement | | After replacement | |
|  |  | R^2^ | RMSE | R^2^ | RMSE |
| Concentration | Slope, Elevation, Soil pH,  Population density | 0.67 | 1.01 | 0.74 | 0.88 |
| Diffusive flux | Slope, Elevation, Artificial proportion,  Groundwater table | 0.62 | 1.07 | 0.67 | 1.01 |

**Table S4 National nitrogen reduction ratios under the Sustainable scenario. Data source:** [**Zhou, Zhang [23]**](#_ENREF_23)

| **Country** | **Ratio (%)** | **Country** | **Ratio (%)** | **Country** | **Ratio (%)** | **Country** | **Ratio (%)** |
| --- | --- | --- | --- | --- | --- | --- | --- |
| Afghanistan | 44 | Dominica | -53 | Malaysia | 11 | Singapore | 47 |
| Albania | 67 | Dominican Republic | 50 | Maldives | 54 | Slovakia | 55 |
| Algeria | 2 | Ecuador | 24 | Mali | 12 | Slovenia | 50 |
| Angola | -51 | Egypt | 27 | Malta | 52 | South Africa | -10 |
| Antigua and Barbuda | -1 | El Salvador | 48 | Mauritius | 22 | Spain | 66 |
| Argentina | 32 | Eritrea | 37 | Mexico | 32 | Sri Lanka | 38 |
| Armenia | 60 | Estonia | 67 | Mongolia | 68 | Sudan | -11 |
| Australia | 55 | Ethiopia | 41 | Montenegro | 65 | Suriname | -64 |
| Austria | 61 | Fiji | 48 | Morocco | 32 | Sweden | 44 |
| Azerbaijan | 62 | Finland | 50 | Mozambique | -40 | Switzerland | 60 |
| Bahamas | 16 | France | 61 | Myanmar | 18 | Syrian Arab Republic | 20 |
| Bahrain | -2 | French Polynesia | 36 | Namibia | 43 | Tajikistan | 67 |
| Bangladesh | 39 | Gabon | -58 | Nauru | 59 | Thailand | 45 |
| Barbados | 31 | Gambia | -36 | Nepal | 40 | Togo | -26 |
| Belarus | 73 | Georgia | 48 | Netherlands | 57 | Tonga | 82 |
| Belgium | 68 | Germany | 63 | New Caledonia | 30 | Trinidad and Tobago | 40 |
| Belize | 23 | Ghana | -209 | New Zealand | 52 | Tunisia | 30 |
| Benin | -35 | Greece | 56 | Nicaragua | 53 | Turkey | 58 |
| Bermuda | 6 | Guatemala | 37 | Niger | 40 | Uganda | 29 |
| Bhutan | 4 | Guinea | -69 | Nigeria | -166 | Ukraine | 24 |
| Bolivia (Plurinational State) | -40 | Guyana | -12 | North Macedonia | 78 | United Arab Emirates | -8 |
| Bosnia and Herzegovina | 71 | Honduras | 33 | Norway | 56 | United Kingdom of Great Britain and Northern Ireland | 56 |
| Botswana | -1 | Hungary | 62 | Oman | 24 | United Republic of Tanzania | -19 |
| Brazil | 19 | Iceland | 72 | Pakistan | 68 | United States of America | 46 |
| Brunei Darussalam | 54 | India | 46 | Panama | 51 | Uruguay | 67 |
| Bulgaria | 62 | Indonesia | 29 | Papua New Guinea | -25 | Uzbekistan | 58 |
| Burkina Faso | 34 | Iran (Islamic Republic of) | 61 | Paraguay | 1 | Venezuela (Bolivarian Republic of) | 13 |
| Burundi | -32 | Iraq | 43 | Peru | 11 | Viet Nam | 21 |
| Cambodia | -21 | Ireland | 82 | Philippines | -9 | Yemen | 24 |
| Cameroon | -59 | Israel | 18 | Poland | 59 | Zambia | -32 |
| Canada | 37 | Italy | 64 | Portugal | 55 | Zimbabwe | 30 |
| Central African Republic | -22 | Jamaica | 28 | Qatar | 15 |  |  |
| Chile | 49 | Japan | 35 | Republic of Korea | 26 |  |  |
| China | 39 | Jordan | 17 | Republic of Moldova | 49 |  |  |
| Colombia | 39 | Kazakhstan | 56 | Romania | 59 |  |  |
| Congo | -63 | Kenya | 13 | Russian Federation | 31 |  |  |
| Cook Islands | 97 | Kuwait | 10 | Rwanda | -70 |  |  |
| Costa Rica | 57 | Kyrgyzstan | 34 | Saint Kitts and Nevis | 25 |  |  |
| Côte d'Ivoire | -104 | Latvia | 70 | Saint Lucia | 24 |  |  |
| Croatia | 51 | Lebanon | 47 | Samoa | 62 |  |  |
| Cuba | 50 | Libya | 22 | Saudi Arabia | 29 |  |  |
| Cyprus | 54 | Lithuania | 67 | Senegal | 7 |  |  |
| Czechia | 64 | Luxembourg | 57 | Serbia | 62 |  |  |
| Democratic Republic of the Congo | -109 | Madagascar | -21 | Serbia and Montenegro | 0 |  |  |
| Denmark | 81 | Malawi | -117 | Seychelles | 25 |  |  |

**Table S5 The proportion of NH_4_^+^-N and NO_3_^-^-N from different sources (percentage of NH_4_^+^-N and NO_3_^-^-N forms in TN)**

| **N sources** | **Proportion of NH_4_^+^-N (%)** | **Proportion of NO_3_^-^-N (%)** | **Reference** |
| --- | --- | --- | --- |
| Atmospheric deposition | 35 | 35 | [[24](#_ENREF_24),[25](#_ENREF_25)] |
| Aquaculture | 15 | 35 | [[26](#_ENREF_26),[27](#_ENREF_27)] |
| Sewage | 74 | 19 | [[28](#_ENREF_28)] |
| Surficial runoff | 10 | 70 | [[29](#_ENREF_29),[30](#_ENREF_30)] |
| Soil loss | 0 | 0 | [[19](#_ENREF_19)] |
| Groundwater exfiltration | 5 | 45 | [[29](#_ENREF_29)] |
| Vegetation in floodplains | 0 | 0 | [[19](#_ENREF_19)] |

**References**

1. Wang G, Xia X, Liu S *et al.* Intense methane ebullition from urban inland waters and its significant contribution to greenhouse gas emissions. *Water Res*. 2021; **189**: 116654.

2. Johnson KM, Hughes JE, Donaghay PL *et al.* Bottle-calibration static head space method for the determination of methane dissolved in seawater. *Analytical Chemistry*. 1990; **62**: 2408-12.

3. Wanninkhof R. Relationship between wind-speed and gas-exchange over the ocean. *Journal of Geophysical Research: Oceans*. 1992; **97**: 7373-82.

4. Wiesenburg DA and Guinasso Jr NL. Equilibrium solubilities of methane, carbon monoxide, and hydrogen in water and sea water. *Journal of Chemical Engineering Data*. 1979; **24**: 356-60.

5. Campeau A, Lapierre JF, Vachon D *et al.* Regional contribution of CO_2_ and CH_4_ fluxes from the fluvial network in a lowland boreal landscape of Quebec. *Global Biogeochemical Cycles*. 2014; **28**: 57-69.

6. Pajala G, Rudberg D, Galfalk M *et al.* Higher Apparent Gas Transfer Velocities for CO_2_ Compared to CH_4_ in Small Lakes. *Environ Sci Technol*. 2023; **57**: 8578-87.

7. Ulseth AJ, Hall RO, Canadell MB *et al.* Distinct air-water gas exchange regimes in low- and high-energy streams. *Nature Geoscience*. 2019; **12**: 259-63.

8. Kuss J and Schneider B. Chemical enhancement of the CO_2_ gas exchange at a smooth seawater surface. *Mar Chem*. 2004; **91**: 165-74.

9. Lin P, Pan M, Beck HE *et al.* Global Reconstruction of Naturalized River Flows at 2.94 Million Reaches. *Water Resources Research*. 2019; **55**: 6499-516.

10. Yamazaki D, Ikeshima D, Sosa J *et al.* MERIT Hydro: A high-resolution global hydrography map based on latest topography dataset. *Water Resources Research*. 2019; **55**: 5053-73.

11. Raymond PA, Hartmann J, Lauerwald R *et al.* Global carbon dioxide emissions from inland waters. *Nature*. 2013; **503**: 355-9.

12. Liu S, Kuhn C, Amatulli G *et al.* The importance of hydrology in routing terrestrial carbon to the atmosphere via global streams and rivers. *Proceedings of the National Academy of Sciences of the United States of America*. 2022; **119**: e2106322119.

13. Carabajal CC, Harding DJ, Boy JP *et al.* Evaluation of the Global Multi-Resolution Terrain Elevation Data 2010 (GMTED2010) Using ICESat Geodetic Control. *International Symposium on Lidar and Radar Mapping 2011: Technologies and Applications*. 2011; **8286**.

14. Cumming GS, Buerkert A, Hoffmann EM *et al.* Implications of agricultural transitions and urbanization for ecosystem services. *Nature*. 2014; **515**: 50-7.

15. Liyanage CP and Yamada K. Impact of population growth on the water quality of natural water bodies. *Sustainability*. 2017; **9**: 1405.

16. He B, Kanae S, Oki T *et al.* Assessment of global nitrogen pollution in rivers using an integrated biogeochemical modeling framework. *Water Res*. 2011; **45**: 2573-86.

17. Xu Z, Jiao L, Lan T *et al.* Mapping hierarchical urban boundaries for global urban settlements. *International Journal of Applied Earth Observation and Geoinformation*. 2021; **103**: 102480.

18. Wang J, Zhao Y, Li C *et al.* Mapping global land cover in 2001 and 2010 with spatial-temporal consistency at 250 m resolution. *ISPRS Journal of Photogrammetry and Remote Sensing*. 2015; **103**: 38-47.

19. Vilmin L, Mogollon JM, Beusen AHW *et al.* Forms and subannual variability of nitrogen and phosphorus loading to global river networks over the 20th century. *Global Planet Change*. 2018; **163**: 67-85.

20. Beusen AHW, Bouwman AF, Van Beek LPH *et al.* Global riverine N and P transport to ocean increased during the 20th century despite increased retention along the aquatic continuum. *Biogeosciences*. 2016; **13**: 2441-51.

21. Raymond PA, Zappa CJ, Butman D *et al.* Scaling the gas transfer velocity and hydraulic geometry in streams and small rivers. *Limnology and Oceanography: Fluids and Environments*. 2012; **2**: 41-53.

22. Stanley EH, Loken LC, Casson NJ *et al.* GRiMeDB: The global river database of methane concentrations and fluxes. *Earth System Science Data Discussions*. 2022; **15**: 2879–926.

23. Zhou Y, Zhang X, Zou Y *et al.* Cutting global nitrogen emissions by one-third for balanced and achievable SDGs by 2030. *One Earth*. 2026; **9**: 101562.

24. Williams MW, Hood E and Caine N. Role of organic nitrogen in the nitrogen cycle of a high-elevation catchment, Colorado Front Range. *Water Resources Research*. 2001; **37**: 2569-81.

25. Duce RA, LaRoche J, Altieri K *et al.* Impacts of atmospheric anthropogenic nitrogen on the open ocean. *Science*. 2008; **320**: 893-7.

26. Bouwman AF, Beusen AHW, Overbeek CC *et al.* Hindcasts and Future Projections of Global Inland and Coastal Nitrogen and Phosphorus Loads Due to Finfish Aquaculture. *Rev Fish Sci*. 2013; **21**: 112-56.

27. Koçer MAT, Kanyilmaz M, Yilayaz A *et al.* Waste loading into a regulated stream from land-based trout farms. *Aquaculture Environment Interactions*. 2013; **3**: 187-95.

28. Krasner SW, Westerhoff P, Chen B *et al.* Impact of wastewater treatment processes on organic carbon, organic nitrogen, and DBP precursors in effluent organic matter. *Environ Sci Technol*. 2009; **43**: 2911-8.

29. Salemi LF, Groppo JD, Trevisan R *et al.* Nitrogen dynamics in hydrological flow paths of a small tropical pasture catchment. *Catena*. 2015; **127**: 250-7.

30. Kim G, Chung S and Lee C. Water quality of runoff from agricultural-forestry watersheds in the Geum River Basin, Korea. *Environ Monit Assess*. 2007; **134**: 441-52.
